# Simulation vs. Reality: A Comparison of In Silico Distance Predictions with DEER and FRET Measurements

**DOI:** 10.1371/journal.pone.0039492

**Published:** 2012-06-25

**Authors:** Daniel Klose, Johann P. Klare, Dina Grohmann, Christopher W. M. Kay, Finn Werner, Heinz-Jürgen Steinhoff

**Affiliations:** 1 Department of Physics, University of Osnabrück, Osnabrück, Germany; 2 RNAP Laboratory, Institute of Structural and Molecular Biology, Division of Biosciences, University College London, London, United Kingdom; 3 Institute of Structural and Molecular Biology, Division of Biosciences, University College London, London, United Kingdom; 4 London Centre for Nanotechnology, University College London, London, United Kingdom; Uni. of South Florida, United States of America

## Abstract

Site specific incorporation of molecular probes such as fluorescent- and nitroxide spin-labels into biomolecules, and subsequent analysis by Förster resonance energy transfer (FRET) and double electron-electron resonance (DEER) can elucidate the distance and distance-changes between the probes. However, the probes have an intrinsic conformational flexibility due to the linker by which they are conjugated to the biomolecule. This property minimizes the influence of the label side chain on the structure of the target molecule, but complicates the direct correlation of the experimental inter-label distances with the macromolecular structure or changes thereof. Simulation methods that account for the conformational flexibility and orientation of the probe(s) can be helpful in overcoming this problem. We performed distance measurements using FRET and DEER and explored different simulation techniques to predict inter-label distances using the Rpo4/7 stalk module of the *M. jannaschii* RNA polymerase. This is a suitable model system because it is rigid and a high-resolution X-ray structure is available. The conformations of the fluorescent labels and nitroxide spin labels on Rpo4/7 were modeled using *in vacuo* molecular dynamics simulations (MD) and a stochastic Monte Carlo sampling approach. For the nitroxide probes we also performed MD simulations with explicit water and carried out a rotamer library analysis. Our results show that the Monte Carlo simulations are in better agreement with experiments than the MD simulations and the rotamer library approach results in plausible distance predictions. Because the latter is the least computationally demanding of the methods we have explored, and is readily available to many researchers, it prevails as the method of choice for the interpretation of DEER distance distributions.

## Introduction

A mechanistic understanding of complex biological systems requires information about their structure and dynamics. Structure determination by X-ray crystallography, NMR and cryoelectron microscopy (cryo-EM) has become indispensable for characterizing multi-subunit enzymes such as RNA polymerases and the ribosome. Probe-based techniques including double electron-electron resonance (DEER) and Förster resonance energy transfer (FRET) spectroscopy are particularly advantageous when describing conformational changes because they are solution techniques. Both methods permit the measurement of intra- and intermolecular distances in the Angström to nanometer range, which makes them ideally suited to garner information about the topology of biomolecules and macromolecular complexes. Neither approach is limited by the size or molecular weight of the system and both are able to provide information on problematic targets such as flexible, less ordered regions [1]–[3] even in native membranes [4]. Hence, DEER and FRET can give valuable insights into the dynamics of a molecular process along a reaction pathway or in response to defined stimuli, while the measurement of changes of inter-probe distances is the most straightforward approach for detecting the conformational dynamics of macromolecules within mobile regions.

FRET is the distance dependent non-radiative energy transfer between a donor and an acceptor fluorophore that occurs if the fluorophores are in close proximity and the emission spectrum of the donor and the excitation spectrum of the acceptor overlap [5]. Due to the development of photostable and bright fluorophores, highly sensitive fluorescence spectrometers and convenient labeling protocols, FRET has wide applications both *in vivo* and *in vitro*. The range of donor-acceptor pairs commercially available allows distance measurements in the range of 25–60 Å, extending up to 100 Å in favorable cases. Single molecule FRET measurements have been proven to be invaluable to determine the architecture of complexes that have resisted crystallographic approaches [6], and to identify and distinguish between diverse conformational subpopulations [7], [8]. Apart from the high intrinsic sensitivity of fluorescence-based experiments, FRET can be performed on freely diffusing molecules in solution; both points are perceived as major advantages over DEER.

DEER spectroscopy takes advantage of paramagnetic centers that are either naturally present in biomacromolecules (e.g. metal ions such as copper [9] or organic cofactors such as flavins [10]) or, more commonly, are site-specifically incorporated by site-directed spin labeling techniques [11], [12]. In the most widely used approach, a cysteine residue is incorporated into the protein at the desired site and subsequently conjugated to a nitroxide spin label. Distances in the range of 5 to 80 Å are determined by measuring the dipole-dipole coupling between two paramagnetic centers in frozen solution with continuous-wave (for distances below 20 Å) or pulsed electron paramagnetic resonance (EPR) techniques such as DEER (for distances above 20 Å) [3], [4], [12].

A key difference between the two techniques is that for DEER spectroscopy the two interacting labels can be identical, which simplifies the labeling strategy, particularly for multimeric proteins, whereas for FRET, donor and acceptor probes are required. Therefore, the labeling strategy is most often reliant on separate incorporation into different polypeptides or nucleic acids before complex formation.

Fluorescence and nitroxide spin labels both exhibit conformational flexibility due to the structure of the linkers by which they are attached to the biomolecule. This property minimizes the influence of the label side chain on the biomolecule conformation, but complicates the accurate prediction of inter-label distances.

Nitroxide spin labels have also found a role in solution NMR spectroscopy with paramagnetic relaxation enhancement (PRE) as a valuable tool for obtaining long-range distance constraints alongside NOE measurements. For obtaining accurate PRE distance restraints again detailed knowledge about the location of the spin label side chain is necessary [13].

**Figure 1:**
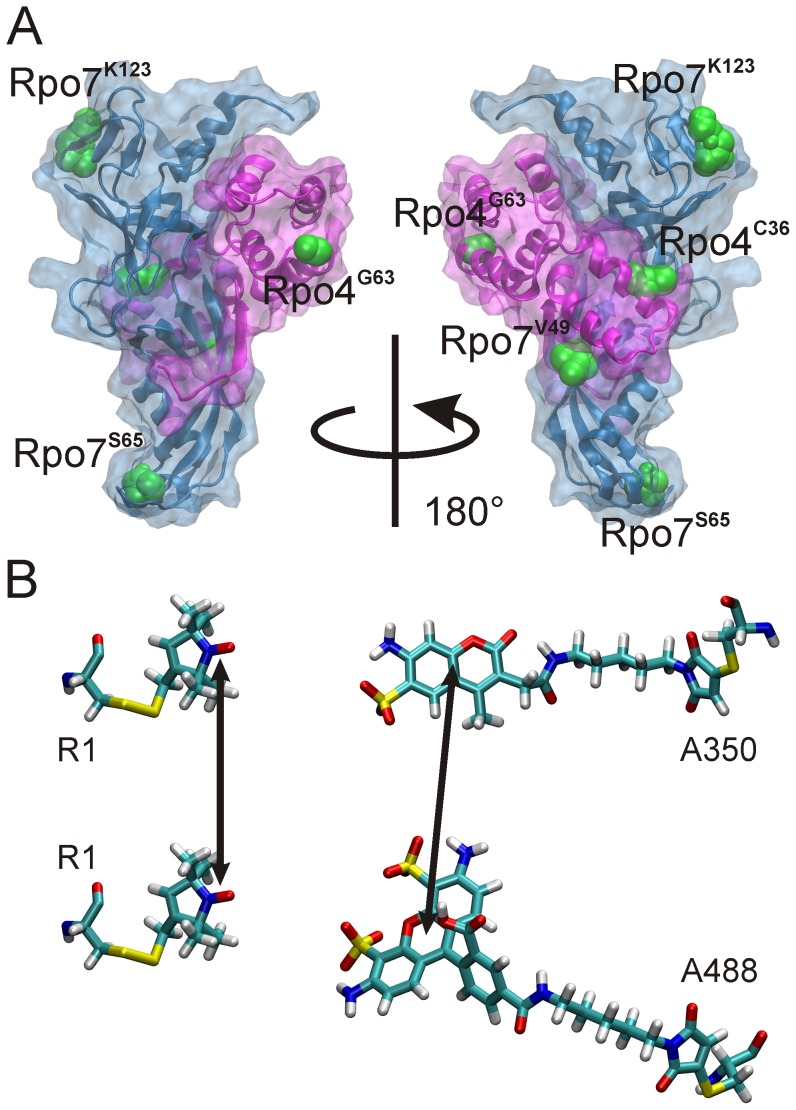
The model system. (A) Crystal structure of the Rpo4/7 complex (pdb: 1GO3) with the positions used for labeling indicated by a spacefill representation of the native side chains (green). (B) Structures of a spin label pair (left) and the fluorophore pair used in this study. The arrows indicate where the electronic orbitals are between which inter label distances are measured. In first approximation for spin labels this is the center of the nitroxide N-O bond, for fluorophores the center of the chromophore region.

Simulation methods are therefore required that account for the linker flexibility and enable a reliable positional modeling of the fluorophore for FRET or spin label probe positions for EPR and NMR. In this study we combine experimental distance measurements by DEER and FRET with three simulation approaches, namely molecular dynamics (MD) simulations, Monte Carlo (MC) conformational search and rotamer library analysis (RLA). We compare the experimental distance data and the ability of the respective simulation techniques to account for linker length and flexibility of the label side chains. The study was performed on the RNA polymerase (RNAP) subunits Rpo4 and Rpo7 (or subunits F and E, respectively) (Figure 1A) from the hyperthermophilic archaeon *Methanocaldococcus jannaschii* [14], [15]. The heterodimeric Rpo4/7 complex is a versatile and suitable model system for the current study because the subunits Rpo4 and 7 can be expressed, purified and labeled individually, and subsequently dimerized to form a fully active subcomplex of the RNAP [16]. The crystal structure of the *M. jannaschii* Rpo4/7 complex has been determined at high resolution and serves as a precise reference point for the FRET and DEER distance measurements. The validity and accuracy of our reference structure is ascertained by the excellent structural alignment of multiple structures of this complex from three archaeal and three eukaryotic species, either as ‘free’ complexes or integrated into the complete RNAP structure (Figure S1). We have previously shown that interactions of the Rpo4/7 complex with its biologically relevant ligand – transcript RNA – do not lead to conformational changes of Rpo4/7 [16]. This indicates that the structure is rigid and implies that it is little prone to crystal packing artifacts. Finally, the X-ray structure enabled the prediction of a RNA ligand binding site that was convincingly confirmed by a molecular genetics analysis – connecting structure to biological function [17]. In summary, the Rpo4/7 crystal reference structure is extremely likely to reflect the solution structure at ambient temperatures as well as in the frozen state.

We introduced fluorescent or spin probes at two positions in Rpo4 (36 and 63) and at three positions in Rpo7 (V49C, S65C and K123C), and carried out distance measurements using FRET and DEER. The results were used to analyze the predictions obtained by several simulation approaches: molecular dynamics; stochastic Monte Carlo sampling; and for spin labels a rotamer library analysis.

## Results

### Characterization of the Labeled Rpo4/7 Derivatives

In order to engineer spin labels or fluorescence labels into either Rpo4 or 7 we introduced single cysteine mutations at various positions of the proteins. We chose positions that according to the structure (Figure 1A), biochemical studies and sequence alignments: i) are surface exposed, ii) show a low degree of conservation, iii) are not close to the proposed RNA binding site, and iv) ideally are located in loop regions of the protein and therefore do not alter any secondary structure elements.

The single cysteine variants were individually labeled either with the fluorophores Alexa350, Alexa488 or the spin label MTSSL (Figure 1B). Spin label side chains are denoted with the additional superscript R1, e.g. Rpo4^G63R1^ for Rpo4, where G63 has been mutated to cysteine and subsequently spin labeled. Fluorophores bound to the protein are denoted with ^*fluorophore^, e.g. Rpo7^S65*A350^ for the S65C mutant of Rpo7, labeled with Alexa Fluor A350.

In all cases the proteins were able to adopt a fold that allows heterodimer formation. To ensure that the proteins folded correctly we tested the functionality of Rpo4/7 in various assays and found that Rpo4/7 dimers were heat stable, able to bind RNA and interact with the RNAP core [16]. These results indicate that modified Rpo4/7 retains the native structure and that the labels do not compromise the activity of the protein.

### Distance Determination by FRET

**Figure 2:**
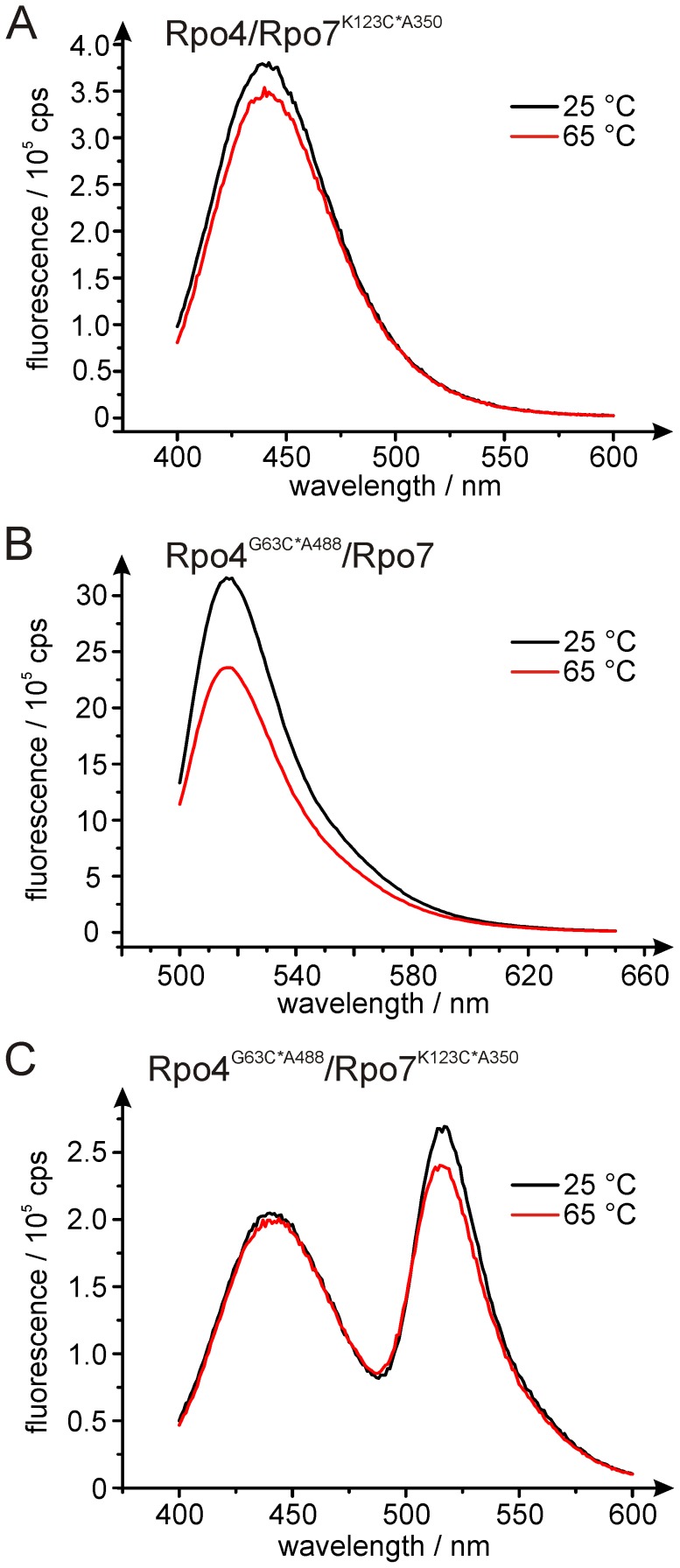
Influence of temperature on the emission intensity of fluorophores. Fluorescence emission spectra at 25°C (black line) or 65°C (red line) are shown for Rpo4/7 samples (50 nM) labeled with (A) donor only (Rpo4/Rpo7^K123C*A350^, excitation at 320 nm), (B) acceptor only (Rpo4^G63C*A488^/Rpo7, excitation at 493 nm) or (C) donor and acceptor (Rpo4^G63C*A488^/Rpo7^K123C*A350^, excitation at 320 nm).

**Figure 3:**
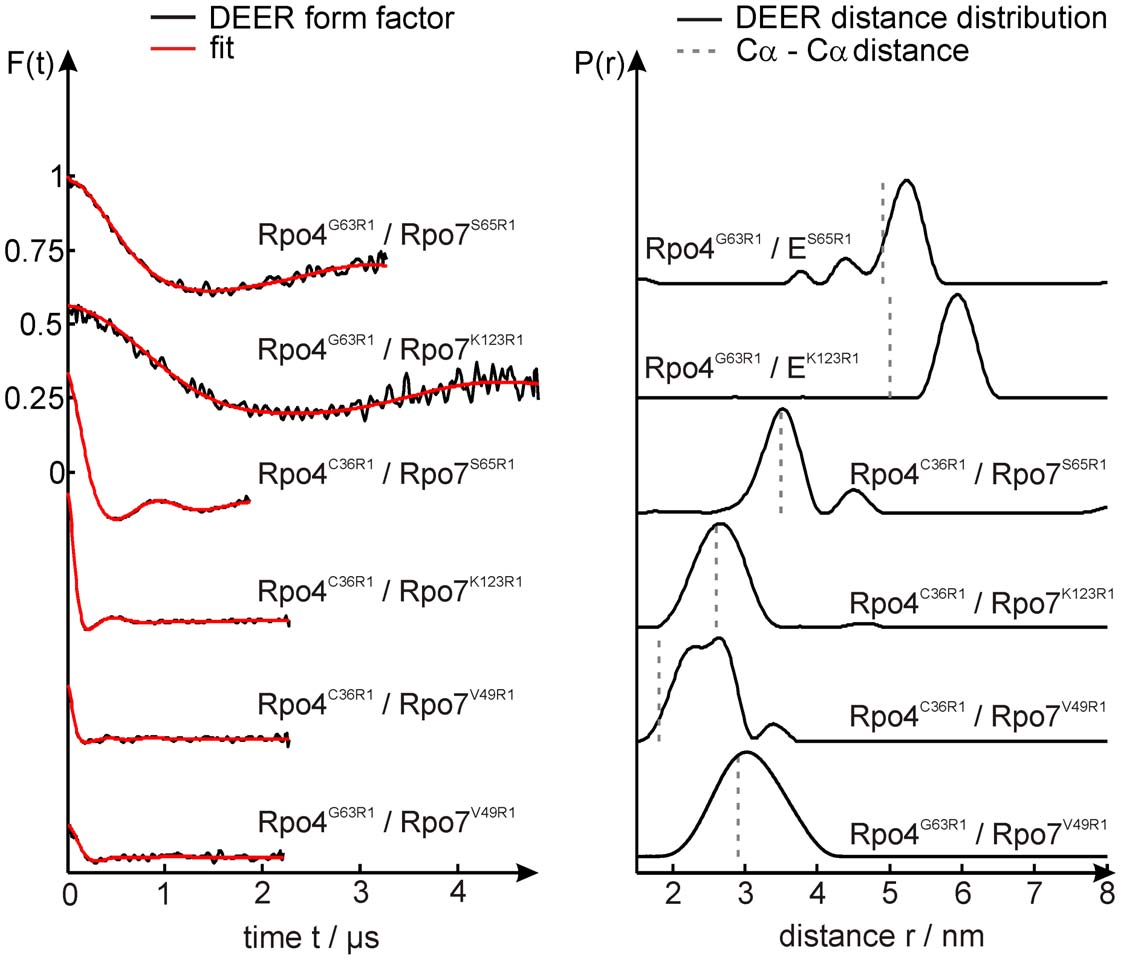
DEER data and distance distributions of doubly spin-labeled Rpo4/7 complexes. Left: background corrected dipolar evolution data; right: distance distributions obtained by Tikhonov regularization. Red traces in the left panel represent the fits obtained by Tikhonov regularization. In the distance distributions Cα-Cα distances derived from the Rpo4/7 crystal structure (pdb: 1GO3) are indicated by gray dashed lines.

The emission spectra of the single donor (D)- or acceptor (A) fluorophore labeled Rpo4/7 complexes (Figure 2A, 2B) show the expected emission maxima typical for the chosen fluorophores (A350∶442 nm, A488∶519 nm). For the donor-acceptor (D-A) labeled dimer (Figure 3C) we detected the expected decrease in donor emission, indicating energy transfer.

Since the Rpo4/7 complex is derived from a hyperthermophilic organism it is important to probe its structure by measuring the emission spectra not only at 25°C but also at an elevated, biologically relevant, temperature of 65°C. Even though the emission maxima are unchanged, the fluorescence intensity decreases by 8% for the donor (A350) and 25% for the acceptor (A488) at 65°C. This behavior can be explained either by higher contact quenching rates or an increase in non-radiative decay rates (e.g. internal conversion) due to increased torsional mobility of the dye, as it has been shown for rhodamine B [18], [19]. Therefore we calculated the FRET efficiencies for the datasets collected at 25°C and 65°C from the decrease in donor fluorescence intensity (see Materials and Methods), which appeared to be less affected by higher temperatures.

The data obtained from the fluorescence experiments with different fluorophore-labeled Rpo4/7 constructs are summarized in Table 1. We found that the transfer efficiency correlates with the distance predicted by the Rpo4/7 structure. Calculation of the inter-fluorophore distances based on the transfer efficiencies (see Materials and Methods) reveals that the separation between Rpo4^G63*A488^ and Rpo7^S65*A350^ as well as Rpo7^K123*A350^ are in good agreement with the Cα-Cα distances obtained from the X-ray structure [20], but the comparatively short distance determined between positions Rpo4^G63*A488^ and Rpo7^V49*A350^ profoundly deviates from the distance derived from the crystal structure (Cα-Cα: 45 vs. 29 Å). The distances calculated from the data collected at 65°C appear slightly larger compared to those obtained at 25°C (by up to 4 Å for Rpo4^G63*A488^ - Rpo7^K123*A350^). However the data show that higher temperatures only marginally influence the FRET efficiency. Hence, FRET provides the opportunity to measure distances and changes thereof in thermophilic systems at elevated temperatures.

**Table 1:** Anisotropy and distance data from ensemble FRET measurements carried out at 25°C and 65°C.

pair	Anisotropy				FRET efficiencies & Distances				
	25°C		65°C		FRET (25°C)		FRET (65°C)		X-ray
	A350	A488	A350	A488	E_FRET_	R_DA_ (Å)	E_FRET_	R_DA_ (Å)	R_Cα-Cα_ (Å)
Rpo4^wt^- Rpo7^V49*A350^	0.112	–	0.072	–	–	–	–	–	–
Rpo4^G63*A488^- Rpo7^V49C^	–	0.114	–	0.070	–	–	–	–	–
Rpo4^G63*A488^- Rpo7^V49*A350^	0.135	0.116	0.085	0.067	0.68	44	0.66	45	29
Rpo4^G63*A488^- Rpo7^S65*A350^	0.110	0.115	0.062	0.062	0.42	53	0.37	55	49
Rpo4^G63*A488^- Rpo7^K123*A350^	0.095	0.115	0.048	0.064	0.38	54	0.28	59	50

The inter-fluorophore distances obtained by determination of the FRET efficiencies and calculation using equation (2) (see Materials and Methods) are compared to the respective Cα-Cα distances from the crystal structure (pdb: 1GO3).

In general, several aspects might contribute to the discrepancy between the Cα-Cα distance and the experimentally determined distance observed for Rpo4^G63*A488^ - Rpo7^V49*A350^. First, the transfer efficiency *E_FRET_* strongly depends on the Förster radius *R_0_* for a particular donor-acceptor pair as well as on the distance between the two fluorophores (see equation 2). Therefore, in general, measurements of the distance *r* are only reliable when *r* is in the range of 0.5 *R_0_*–1.5 *R_0_*. Here, we chose a D-A pair with a *R_0_* = 50 Å – the shortest one available for Alexa fluorophores – so that all chosen Rpo4-Rpo7 distances should be within the 0.5–1.5 *R_0_* range. Nevertheless, the Cα-Cα distance for Rpo4^G63^– Rpo7^V49^ of 29 Å might bring the two dyes within a distance <25 Å = 0.5 *R_0_.* The importance of choosing a D-A pair with the appropriate distance boundaries becomes even more evident when using a D-A pair with a larger Förster radius. Using the A488–A594 pair with *R_0_* = 60 Å, the distance determined between positions Rpo4^G63^ and Rpo7^V49^ was 65 Å (data not shown). Second, FRET in the short distance range might be complicated by the presence of additional fluorescence quenching pathways that reduce the donor emission other than by energy transfer to the acceptor [21], [22]. Third, according to Förster’s theory [23], *R_0_* depends on the relative orientation of the two dyes, expressed in the orientation factor κ^2^ (equation 3). Therefore, κ^2^ is a major determinant of the distance predicted from FRET [24]. For freely rotating donor and acceptor pairs, a value for the orientation factor of 2/3 can be assumed [25]. Fluorescence anisotropy measurements and molecular dynamic simulations can indicate whether or not donor and acceptor are randomly orientated. In general, a fluorescence anisotropy of less than 0.2 is normally assigned to a κ^2^ value of 2/3 [26]. We tested this aspect for our system and found that donor and acceptor anisotropies are clearly below the limit of 0.2 ranging from 0.095 to 0.135 (25°C), indicating that the dyes are able to rotate freely (Table 1). Measured at 65°C the anisotropy decreases further to values between 0.048 and 0.085. These data suggest that the use of an orientation factor of 2/3 is applicable in our model system and consequently influences of the relative orientations of the fluorophores on the experimental distances can be safely neglected. Finally, and of crucial importance even when the previous aspects can be neglected, it has to be considered that, due to the length of the linker between the protein and the optical center of the fluorophore (Figure 2B), large deviations of the measured distances from the Cα-Cα distances derived from a crystal structure might occur. Clegg and co-workers for example estimated the standard deviation of the inter-dye distance caused by the flexible linker for an A488–A568 pair to be ∼7.4 Å from the Cα-Cα distance [27]. In this study, we will address this issue by applying two simulation techniques, namely MD simulations and a MC conformational search to account for the linker structure and flexibility. The results of the simulations for the FRET pair used here will be presented and discussed in the respective sections.

### Distance Determination by DEER

The single cysteine variants (Figure 1A) were modified with the spin label MTSSL (see Figure 1B and Materials and Methods), yielding the spin label side chain R1. The experimental results are shown in Figure 3, where the left panel shows the background-corrected dipolar evolution data and the right panel the corresponding distance distributions obtained by Tikhonov regularization (see Materials and Methods and Text S1). Details of the distance analysis as well as the respective dipolar spectra are given in the supplementary information.

All spin label combinations investigated here exhibit well defined inter spin distance distributions ranging from ∼25 Å (Rpo4^C36R1^/Rpo7^V49R1^) to ∼59 Å (Rpo4^G63R1^/Rpo7^K123R1^) with distribution widths ranging from 6 to 11 Å (Table 2). Comparison of the experimental inter spin distance distributions with the Cα-Cα distances calculated from the Rpo4/7 crystal structure (indicated in the DEER distance distributions in Figure 3 by gray dashed lines) shows agreement (Δr ≤2 Å) in three cases (Rpo4^C36R1^/Rpo7^S65R1^, Rpo4^C36R1^/Rpo7^K123R1^, Rpo4^G63R1^/Rpo7^V49R1^ and Rpo4^G63R1^/Rpo7^S65R1^), but deviations of 7 and 9 Å for the other two spin labeled molecules. As in the case of the fluorophore, residue Cα-Cα distances need not necessarily be identical to the measured distances since the DEER data represent *inter spin* distances. The unpaired electron giving rise to the EPR signal is to a good approximation located between the nitrogen and oxygen atom of the spin label NO group (Figure 1B), and the flexibility of the spin label side chain can cause variations in the distance between the Cβ atom and the NO group in the range of 4 to 8 Å [11]. Consequently, the NO-NO distances obtained from the DEER experiment can differ up to 16 Å from the respective Cβ-Cβ distances and even more from the corresponding Cα-Cα distance, clearly encompassing the deviations observed here.

**Table 2:** Experimental DEER distances compared to distances derived from the crystal structure.

Label pair	X-ray Cα-Cα (Å)	DEER (Å)
Rpo4^C36^- Rpo7^V49^	18	25/9
Rpo4^C36^- Rpo7^S65^	35	36/6
Rpo4^C36^- Rpo7^K123^	26	26/8
Rpo4^G63^- Rpo7^V49^	29	31/11
Rpo4^G63^- Rpo7^S65^	49	51/8
Rpo4^G63^- Rpo7^K123^	50	59/6

Cα-Cα distances are determined from the crystal structure (pdb: 1GO3). DEER represent mean distances (center of gravity of the distance distributions). The second number gives the full width of the distance distribution at half maximum.

### Relating Inter Label Distances to Protein Structure

As expected from the lengths and flexibilities of the respective linker moieties, the distances obtained with the two techniques show variable deviations up to 16 Å for the FRET experiments and 9 Å for the DEER results from the backbone-backbone distances. Remarkably, it has been shown that conversion of inter spin distances into distance ranges between backbone atoms by using a simple “motion-on-a-cone” model in combination with EPR accessibility data and the de novo structure prediction algorithm Rosetta suffice to obtain accurate, atomic-detail models with resolutions approaching 1 Å [28]. For applications which require a more precise relation of probe and backbone positions simulation techniques have to be applied. Calculated inter-label distances can then be compared to the experimental data to evaluate a given structural model or to identify conformational changes. As described in the following sections, we applied different simulation techniques, namely *in vacuo* MD simulations and a MC conformational search for fluorophores, supplemented by MD simulations *in aqua*, i.e. in explicit water, and a rotamer library analysis for spin labels. The latter approach developed by Jeschke and co-workers [29] has not yet been implemented for fluorophores. We compare the results of the different simulation techniques and discuss their applicability with reference to the computational complexity – an important issue, as the expertise and also the infrastructure required for some of the calculations is not necessarily available to all those who want to analyze and interpret FRET or DEER data. As a benchmark for the simulations, the distance distributions are compared in detail for their shape and deviation profile.

### Simulation of FRET Distances

In contrast to inter spin label distances, FRET-derived distance data are not routinely treated with simulation techniques, with only a few attempts in the literature so far [30]–[32]. In this study we carried out *in vacuo* MD and a MC-based conformational search for the fluorophore pair (Alexa350/488) used in the FRET experiments. The MD simulations are performed at 2000 K [32] over 200 ns with constrained positions of backbone carbons and nitrogens. Electrostatic interactions were disabled to further enhance conformational sampling [31]. We did not perform fluorophore label simulations with explicit water at 310 K, as a sufficient conformational sampling of the dyes can be only achieved with simulation times of several microseconds, an exercise not yet manageable using standard computer hardware. Furthermore, as the fluorescence anisotropies in the experiments have been determined to be well below 0.2 (see above), we decided to neglect the influence of the orientation factor on the FRET efficiencies and the derived distances. Consequently, we did not analyze the chromophore orientations in the simulations. Nevertheless, in principle both approaches provide this information which may be used if the experimental fluorescence anisotropy indicates an influence on the FRET efficiencies.

**Figure 4:**
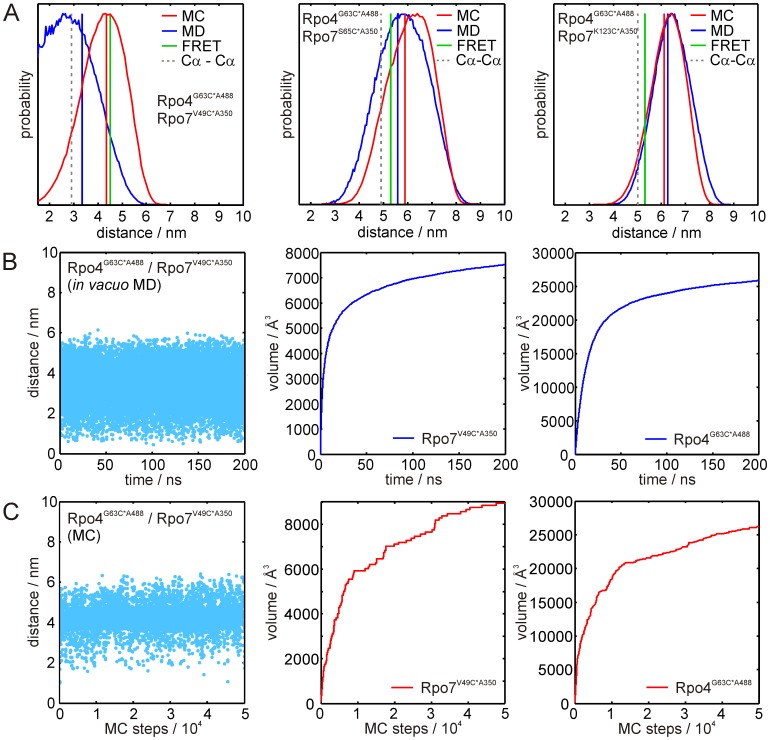
Results of the FRET label simulations. (A) Distance distributions for the label pairs Rpo4^G63C*A488^/Rpo7^V49C*A350^ (left), Rpo4^G63C*A488^/Rpo7^S65C*A350^ (center) and Rpo4^G63C*A488^/Rpo7^K123C*A350^ (right). Distances obtained from the FRET experiments are indicated by green vertical lines, distance distributions obtained from the MD simulations and the MC samplings are shown in blue and red, respectively. Blue and red vertical lines represent expected FRET distances calculated from the respective simulated distance distributions. Cα-Cα distances obtained from the crystal structure are marked by grey lines. (B) Results of the MD simulation for Rpo4^G63C*A488^/Rpo7^V49C*A350^. Left panel: Distance trajectory; center and right panel: Volume sampled by the FRET labels over simulation time for labels Rpo7^V49C*A350^ and Rpo4^G63C*A488^, respectively. (C) Results of the MC sampling for Rpo4^G63C*A488^/Rpo7^V49C*A350^. Left panel: Distance trajectory; center and right panel: Volume sampled by the FRET labels over the number of MC sampling steps for labels Rpo7^V49C*A350^ and Rpo4^G63C*A488^, respectively.

The distance distributions resulting from the *in vacuo* MD simulations (blue) and MC sampling (red) for the FRET pairs are shown in Figure 4A. The corresponding vertical lines are the predicted FRET distances calculated from the simulated distance distributions, taking the 1/r^6^ dependency of the transfer efficiency into account. For comparison, the distances determined from the FRET experiments are indicated as green vertical lines (cf. Table 3). Within the margins given by the simulated distribution widths all simulated mean distances agree statistically with the corresponding experimental values. However, the mean distances calculated from the different methods deviate to different degrees. In detail, the ability of the *in vacuo* MD to reproduce the experimental distances strongly depends on the positions of the fluorophores on the protein. Experimental and calculated distances for the pair Rpo4^G63^/Rpo7^S65^ agree reasonably well (Δr =  +3 Å, simulated *-* experimental distance), whereas for the other two pairs, Rpo4^G63^/Rpo7^V49^ and Rpo4^G63^/Rpo7^K123^, deviations of Δr = −12 Å and +9 Å are observed. In general, three factors can account for such deviations. First, the attached fluorophore might influence the protein structure. This possibility seems unlikely as the function of Rpo4/7 is not impaired and the long linker (Figure 1B) should prevent significant influence of the fluorophore on the protein structure. Second, the force field used in the simulation might not accurately reflect the interactions that determine the motion of the fluorophore, or third, the conformational sampling within the applied simulation time might be incomplete. The latter possibility can be tested by inspection of the volume the fluorophore samples over the simulation time, which should exhibit a convergent (∼asymptotic) behavior. In Figure 4B the distance trajectory and the volumes sampled by the fluorophores are shown for the pair with the highest deviation from the experimental FRET distance, Rpo4^G63^/Rpo7^V49^ (distance trajectories and accessed volumes for the other pairs are shown in Figure S2). For both fluorophores, the expected asymptotic behavior is observed, indicating that conformational sampling within the simulation time of 200 ns is almost complete, unless a high energy barrier prevents one or both of the fluorophores from reaching another conformation with significantly lower energy, i.e. the system is trapped in a local energy minimum due to an inappropriate starting conformation.

**Table 3:** Comparison of experimental and calculated mean distances for the fluorophore pairs.

Label pair	X-ray Cα-Cα (Å)	FRET distance (Å)	MD distance (Å)	MC distance (Å)
Rpo4^G63C*A488^- Rpo7^V49C*A350^	29	45±1	33 (26±17)	43 (37±12)
Rpo4^G63C*A488^- Rpo7^S65C*A350^	49	53±2	56 (58±14)	59 (63±13)
Rpo4^G63C*A488^- Rpo7^K123C*A350^	50	53±5	62 (65±10)	61 (67±9)

Cα-Cα distances are determined from the crystal structure (pdb: 1GO3). FRET distances are derived from the data collected at 25°C, calculated from the simulated distance distributions, taking the 1/r^6^ dependency of the transfer efficiency into account.Values given in brackets are the maxima of the distance distributions. Errors given for the FRET distances represent the standard deviations, those given for the calculated distances from the simulations represent the width of the distance distribution at half maximum.

Problems arising from trapping the system in a local energy minimum are not encountered if stochastic conformational search methods are used, for example in a MC sampling. For each label a trajectory of 50,000 MC sampling steps was generated and inter label distances were obtained non-synchronously using a sliding window as described in Materials and Methods. To test whether 50,000 MC steps are sufficient to sample the accessible conformational space for the fluorescence labels, we carried out additional MC sampling for Rpo4^G63C*A488^/Rpo7^S65C*A350^ with 100,000 MC steps (see Figure S3). The obtained distance distributions and the volumes sampled by the fluorophores are almost identical to those obtained from 50,000 MC sampling steps. The obtained distance distributions either coincide almost perfectly with the MD results (Rpo4^G63^/Rpo7^K123^), or deviate as for Rpo4^G63^/Rpo7^S65^ (Δr (MD – MC) = −3 Å) and for Rpo4^G63^/Rpo7^V49^ (Δr = −10 Å). Strikingly, for the pair that showed the largest difference between MD- and MC-derived distances, MC sampling almost perfectly reproduces the experimental distance (−2 Å). Interestingly, the final accessed volumes for this label pair (Figure 4C) are almost identical to those obtained in the 200 ns *in vacuo* MD simulation. Nevertheless, the conformational space sampled differs significantly between the two approaches, as can be seen from inspection of the label orientation probability distributions (see discussion section and supplementary Figure S4). Whereas the label orientation probabilities for Rpo4^G63^ are almost superimposable, for Rpo7^V49^ it is significantly shifted towards that of Rpo4^G63^ for the MD compared to the MC simulation, leading to the observed differences in the calculated distances. The differences for Rpo7^V49^ result from the fact that in the MD simulation the label side chain is mainly pointing away from the protein surface, caused by the weakness of the van der Waals interactions at 2000 K. In the MC sampling performed at 300 K these attractive forces lead to the label being oriented mainly along the protein surface.

**Table 4:** Comparison of experimental and calculated mean distances for spin label pairs.

Label pair	X-ray Cα-Cα(Å)	DEER (Å)	*in vacuo* MD(Å)	*in aqua* MD(Å)	MC (Å)	RLA (Å)	RLA X1/X2 sel. (Å)
**Rpo4^C36R1^- Rpo7^V49R1^**	18	25/9	30/3	27/6	27/5	28/7	26/7
**Rpo4^C36R1^- Rpo7^S65R1^**	35	36/6	33/9	37/6	38/6	37/8	38/7
**Rpo4^C36R1^- Rpo7^K123R1^**	26	26/8	29/3	26/9	22/9	27/8	28/5
**Rpo4^G63R1^- Rpo7^V49R1^**	29	31/11	26/10	30/10	31/8	31/3	28/6
**Rpo4^G63R1^- Rpo7^S65R1^**	49	51/8	52/7	56/14	53/10	57/7	53/8
**Rpo4^G63R1^- Rpo7^K123R1^**	50	59/6	57/9	61/10	56/9	56/7	59/7

Cα-Cα distances are determined from the crystal structure (pdb: 1GO3). Experimental DEER distances and calculated distances represent mean distances (center of gravity of the distance distributions). The second number gives the full width of the distance distribution at half maximum.

### Simulation of DEER Distance Distributions

MD simulations [33], MC conformational search methods [34], and rotamer library analysis (RLA) [29], [35] are used to simulate inter spin distances. We first consider the MD simulations [33] and MC methods [34], treating the RLA subsequently, as this latter approach does not simulate the dynamic behavior of the spin label side chains but provides probabilities for different rotamer states. We performed *in vacuo* MD simulations at 600 K [36] for 100 ns (with constrained backbone carbon and nitrogen positions) and MD simulations including explicit water at 310 K for 40 ns (without backbone restraints). As for the FRET simulations, the MC sampling was carried out over 50,000 steps.

**Figure 5:**
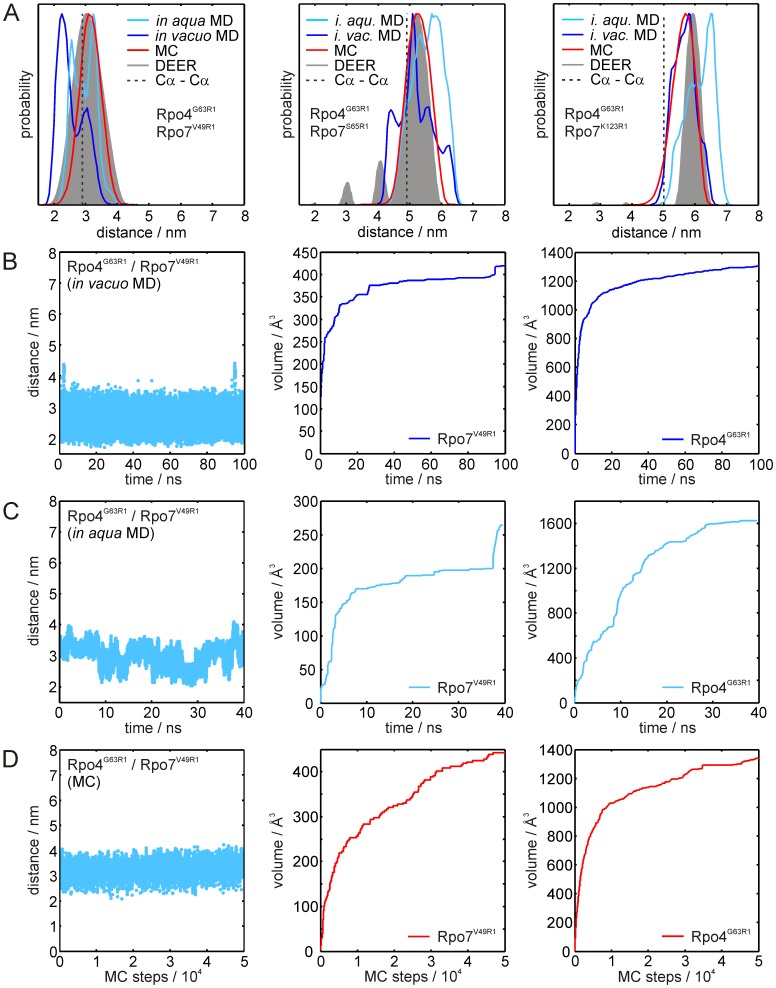
Results of the simulations for spin label pairs Rpo4^G63R1^/Rpo7^xR1^. (A) Distance distributions for the spin label pairs Rpo4^G63R1^/Rpo7^V49R1^ (left), Rpo4^G63R1^/Rpo7^S65R1^ (center) and Rpo4^G63R1^/Rpo7^K123R1^ (right). Distance distributions obtained from the DEER experiments are shown in gray, the results of the *in vacuo* MD, *in aqua* MD and MC simulation are shown in dark blue, cyan and red, respectively. Cα-Cα distances obtained from the crystal structure are marked by gray dashed lines. (B) Results of the *in vacuo* MD simulation for Rpo4^G63R1^/Rpo7^V49R1^. Left panel: Distance trajectory; center and right panel: Volume sampled by the spin labels over simulation time for labels Rpo4^G63R1^ and Rpo7^V49R1^, respectively. (C) Corresponding results for the *in aqua* MD simulation. (D) Results of the corresponding MC samplings. For the *in* vacuo MD simulations and the MC samplings of the other spin label pairs the distance trajectories and volume plots are given in the Supplementary Information, Figure S4. The corresponding data for the *in aqua* MD simulation are given in Figure S5.

The distance distributions obtained from the MD simulations *in vacuo* (blue) and in explicit water (cyan) and the MC sampling approach (red) for the spin label pairs Rpo4^G63R1^/Rpo7^V49R1^, Rpo4^G63R1^/Rpo7^E65R1^ and Rpo4^G63R1^/Rpo7^K123R1^ are shown together with the experimental distance distributions (gray) in Figure 5A. The only statistically significant deviation from the experiments, taking the full widths of the simulated distributions into account, is found for the two *in vacuo* MD distance distributions for Rpo4^C36R1^/Rpo7^V49R1^ and Rpo4^C36R1^/Rpo7^K123R1^ (Table 4).

Nevertheless, close inspection of the distributions reveals additional features. For example, for the pair Rpo4^G63R1^/Rpo7^V49R1^ the *in vacuo* MD simulation yields a distance distribution (Figure 5A, left, in blue) with two maxima, one at 31 Å coinciding almost perfectly with the major peak of the wider experimental distribution, and one at ∼22 Å that is not in accordance with the experiment. The reason for the additional peak becomes clear from the inspection of the distance and volume trajectories shown in Figure 5B and the spatial probability distribution of the labels. The interspin distance trajectory for Rpo4^G63R1^/Rpo7^V49R1^ exhibits continuous rapid jumps of up to ∼20 Å between the two states in the distance distribution and the volume plots show the expected asymptotic behavior, especially for the less restricted position Rpo4^G63R1^. Inspection of the probability distributions for the labels (see discussion section) reveals that these two states localize on either of the two sides of the helix to which the spin label is attached. Such a biphasic spatial distribution present at only one position of a spin label pair clearly leads to a bimodal distance distribution. The comparison with the experimental distribution indicates that either the total energy of the rotamer responsible for the 22 Å distance is underestimated, or the energy of the rotamer corresponding to the 31 Å distance is overestimated. Furthermore, inspection of the distance trajectory also reveals that a small contribution of distances reaching out to 40 Å is present, clearly in accordance with the experimental distribution. A similar yet less pronounced behavior is observed for the MD simulation with explicit water (Figure 5A, left, in cyan). Here, two major peaks are present at distances of 25 Å and 32 Å, both coinciding well with the experimental distance distribution. In this case not only the accessed volume plot for Rpo7^V49R1^ (Figure 5C, right panel) but also that for Rpo4^G63R1^ (center panel) exhibits several jumps, again indicating transitions of the spin label to a formerly unpopulated rotameric state. Accordingly, several jumps can also be seen in the distance trajectory (Figure 5C, left panel). In spite of the incomplete sampling, the overall agreement between simulation and experiment appears to be better for the *in aqua* MD simulation than for the *in vacuo* MD simulation.

Comparison of the volumes sampled by the spin labels in the *in vacuo* and *in aqua* MD simulations for Rpo4^G63R1^ as well as for Rpo7^S65R1^ and Rpo7^K123R1^, but not for Rpo7^V49R1^, reveals that the maximum value found at the end of the simulation is higher for the *in aqua* MD (Figures S5, S7), resulting from the protein dynamics for which only the *in aqua* MD accounts. Although the protein backbone is also kept fixed in the MC sampling, this approach leads to a more complete sampling of the label side chain conformational space and consequently to larger volumes compared to the *in vacuo* MD simulations. Position Rpo7^V49R1^ appears to be an exception. Here, the *in aqua* MD exhibits a final volume that is ∼40% smaller than that found in the MC sampling or *in vacuo* MD simulation leading to the conclusion that conformational sampling of the spin label side chain within 40 ns simulation time is incomplete.

Good agreement between MC sampling and experiment is obtained for Rpo4^G63R1^/Rpo7^V49R1^ (Figure 5A, left, in red). The distance range and the distribution shape coincide almost perfectly: only the maximum of the simulated distance distribution appears to be shifted to larger distances by about 1 Å and its width is 25% smaller. For the pairs Rpo4^G63R1^/Rpo7^S65R1^ (Figure 5A, center panel) and Rpo4^G63R1^/Rpo7^K123R1^ (right panel) the two MD simulations provide peaks coinciding with the maxima in the experimental distance distributions. The major maxima of the simulated distributions, however, do not coincide with the experimental ones. On the other hand almost perfect agreement between simulation and experiment is obtained for the MC sampling of Rpo4^G63R1^/Rpo7^S65R1^. Here, except being slightly broader towards longer distances, the simulated distribution virtually coincides with the experimental result. For Rpo4^G63R1^/Rpo7^K123R1^ *in vacuo* MD simulation and MC sampling yield almost the same distance distribution, which is in better agreement with the experiment than the *in aqua* MD simulation, but appears to be significantly broadened towards shorter distances. The corresponding distance trajectories and volume plots for Rpo4^G63R1^/Rpo7^S65R1^ and Rpo4^G63R1^/Rpo7^K123R1^ are shown in Figure S5.

In conclusion, for the three spin label pairs described above, the MC sampling approach seems to exhibit the best overall performance to reproduce the experimental DEER data. To verify this conclusion, we discuss in the following section the second set of spin label pairs, where Rpo4 was labeled at position C36R1 and combined with the same set of spin labeled cysteine mutants of Rpo7 used above.

**Figure 6:**
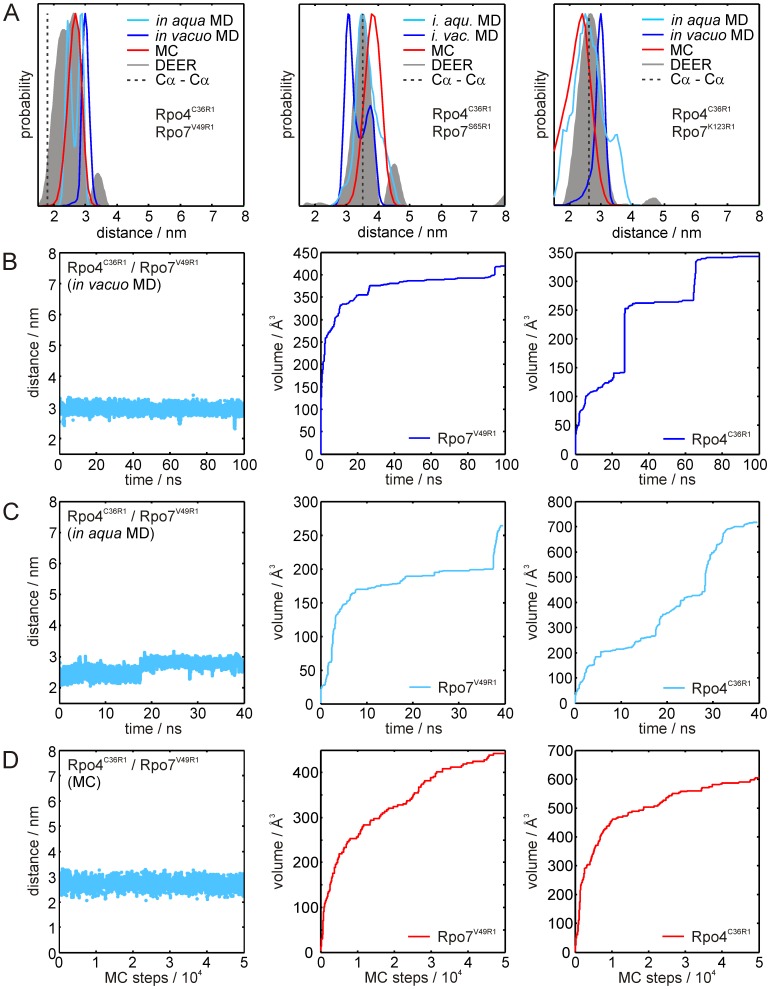
Results of the simulations for spin label pairs Rpo4^C36R1^/Rpo7^xR1^. (A) Distance distributions for the spin label pairs Rpo4^C36R1^/Rpo7^V49R1^ (left), Rpo4^C36R1^/Rpo7^S65R1^ (center) and Rpo4^C36R1^/Rpo7^K123R1^ (right). Distance distributions obtained from the DEER experiments are shown in gray, the results of the *in vacuo* MD, *in aqua* MD and MC simulation are shown in dark blue, cyan and red, respectively. Cα-Cα distances obtained from the crystal structure are marked by gray dashed lines. (B) Results of the *in vacuo* MD simulation for Rpo4^C36R1^/Rpo7^V49R1^. Left panel: Distance trajectory; center and right panel: Volume sampled by the spin labels over simulation time for labels Rpo4^C36R1^ and Rpo7^V49R1^, respectively. (C) Corresponding results for the *in aqua* MD simulation. (D) Results of the corresponding MC samplings. For the *in vacuo* MD simulations and the MC samplings of the other spin label pairs the distance trajectories and volume plots are given in the Supplementary Information, Figure S5. The corresponding data for the *in aqua* MD simulation are given in Figure S6.

The distance distributions for Rpo4^C36R1^/Rpo7^V49R1^ are shown in Figure 6A. The *in vacuo* MD simulation results in a significantly narrower and by ∼5 Å shifted distance distribution. The result from the *in aqua* MD simulation coincides somewhat better with the experiment, but is still slightly shifted (+2 Å), more narrow and moreover bimodal. Here, inspection of the volume plots (Figure 6B and 6C) reveals several jumps, indicating that, especially for Rpo4^C36R1^, transitions to formerly unpopulated rotamers take place. Furthermore, the *in vacuo* MD simulation only reaches half of the total sampled volume of that obtained during the *in aqua* MD simulation; the spatial probability distributions (see discussion section) reveal that only one of the two distinct conformations found by the *in aqua* MD simulations is sampled *in vacuo*. Therefore, although both volume plots show a plateau at the end of the simulation, conformational sampling is still incomplete. Again, the distance predictions provided by the MC sampling approach are in better agreement with the experimental distance distribution, although the shoulder at shorter distances present in the experimental data is not reproduced.

For Rpo4^C36R1^/Rpo7^S65R1^ (Figure 6A, center panel and Figures S6, S7) the *in aqua* MD simulation yields the closest match to the experimental distance distribution. The *in vacuo* MD again exhibits a biphasic behavior for position Rpo7^S65R1^ and shows a bias towards shorter distances and a local minimum in the distance distribution, where the experimental one has its maximum. MC sampling yields a distance distribution with the correct shape and width that is only shifted by ∼3 Å to larger distances.

For Rpo4^C36R1^/Rpo7^K123R1^ (Figure 6A, right panel and Figures S6, S7) the MC sampling and the MD simulation with explicit water perform comparably well. MC sampling yields a distance distribution with the same shape and width as observed in the experiment, but shifted by ∼3 Å to shorter distances. On the other hand, the *in aqua* MD simulation better reproduces the mean distance, yet the overall distance range is wider ranging from 15–40 Å compared to 20–35 Å for the experimental distance distribution. In contrast, the distance distribution from the *in vacuo* MD simulation is significantly narrower than the experimental one and also shifted towards larger distances by ∼3 Å.

**Figure 7:**
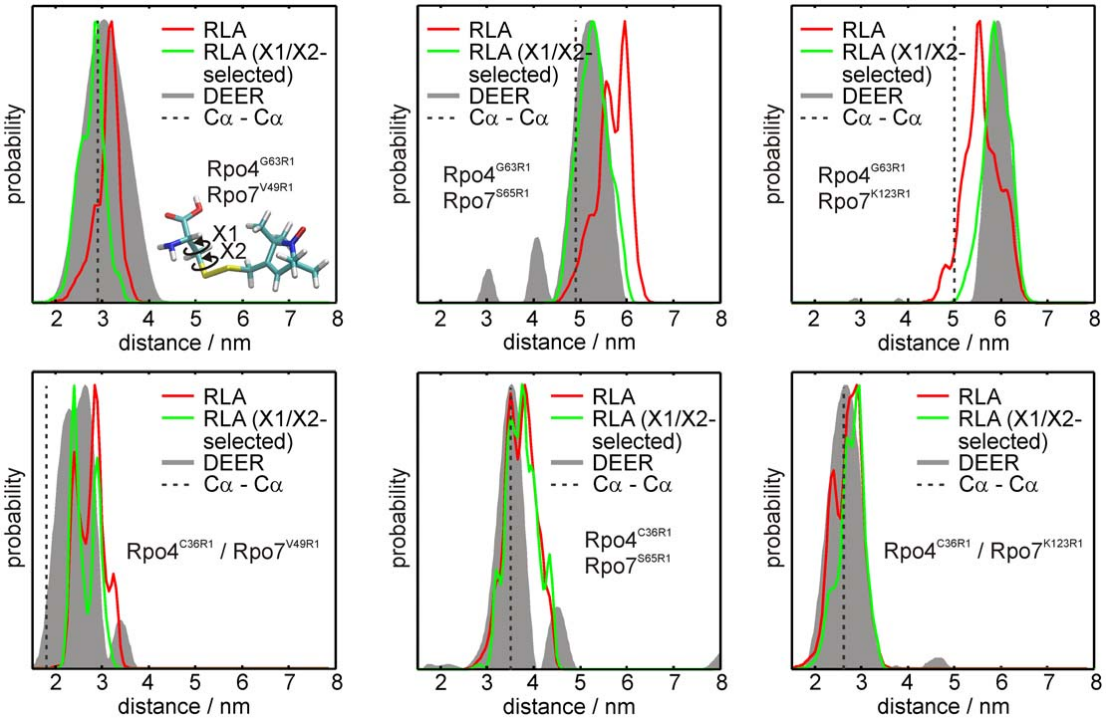
Rotamer library analysis for the spin label pairs in Rpo4/Rpo7. Distance distributions resulting from the DEER experiments are shown in gray. Simulated distance distributions were obtained from the RLA (red) and from a rotamer selection according to the crystal structures of spin labeled T4 lysozyme (green) [37]. The inset in the upper left panel shows the two dihedral angles X1 and X2 discussed in the text (see also Figure S7).

In Figure 7 the distance distributions obtained from the RLA are compared to the experimental results (gray). Although in the RLA side chain and backbone dynamics of the protein are neglected (apart from the “forgive” factor discussed later), the results reveal – especially in comparison to the other simulation techniques (Figures 5 and 6) – that the overall performance in reproducing the experimental distance distributions is remarkably good. The agreement between calculation and experiment can even be further enhanced by taking into account the results of a crystallographic study of R1-labeled T4 lysozyme [37], where Fleissner *et al.* found that R1, when bound to α-helical sites, exhibits mainly three rotamers for the first two dihedral angles, X1 and X2 (see inset in Fig. 7 and Figure S8A), at both cryogenic and ambient temperatures. Only two of those three rotamers ({m,m} and {t,p}, see [37] for the nomenclature used here) are highly populated, possibly due to the formation of a weak intra-spin label hydrogen bond (Cα-Hα···Sδ). Here, for the α-helical positions Rpo4^G63R1^ and Rpo4^C36R1^, we performed a selection of only {m,m} and {t,p} rotamers within the dihedral angle distributions (see Figure S8B). The agreement of the simulated distance distributions with the experimental results improved significantly (Figure 7), leading to almost perfect reproduction of the experimental distributions for all six spin label pairs under investigation. This finding underlines the conclusion made from the comparison between the two MD simulations and the MC sampling approach that protein dynamics can, at least in the cases investigated here, be largely neglected. Since in the RLA the protein structure is kept fixed, a so-called “forgive factor”, that “softens” the interatomic potentials by scaling down the equilibrium interatomic distance in the Lennard-Jones term (see [29] for details), rudimentarily accounts for side chain dynamics in the rotamer energy calculations.

## Discussion

FRET and DEER have been developed and applied successfully to address the structural and dynamic properties of complex biomolecular systems which often are the key for understanding their mechanism. The mobility and orientation of the probes can have a substantial impact on the readout of the experiment. The simulation of fluorescence and spin labels bound to biomolecules not only provides a better understanding of their dynamic behavior, but also allows direct correlation of distance data to structural models. We have compared the results from different simulation techniques for fluorescence and spin labels with experimental distances derived by FRET and DEER using the heterodimeric complex Rpo4/7.

In general, the predicted distances and distance distributions show statistical agreement with the experimental ones for 32 out of 36 examples in terms of the mean distances, and for the distribution widths 17 out of 30 cases match within 2 Å (from the ensemble FRET experiments no distance distribution widths are obtained to be compared to the simulation results). To capture the finer details, we evaluate here the capability of the simulation methods to also predict what is much more challenging, namely the observed shape of the distance distributions.

For the fluorescent label simulations, despite the length of the MD trajectories and the clear convergence of the sampled volume, the MC sampling approach on average performs significantly better in reproducing the experimental FRET distances than the rapid sampling MD simulations at elevated temperature. This becomes more evident if the average distances to be expected in the FRET experiment are calculated from the predicted distance distributions (Table 3, numbers given in brackets). We did not test MD simulations at 310 K *in aqua* for the FRET labels, as the computational effort to calculate MD trajectories sufficiently long to ensure complete conformational sampling (>>200 ns from our estimation based on the *in vacuo* simulations) is exceptionally high, so that such simulations can only be performed using high performance computation facilities. Consequently, a stochastic sampling approach that circumvents trapping of the simulation in local energy minima seems to be the method of choice for the simulation of fluorescence labels comprising long linker moieties and comparably large fluorophores. Ensemble FRET distance measurements combined with standard simulation approaches can provide distance constraints for modeling or validation of structures yet with broad distributions. This limitation can be overcome if multiple single-molecule FRET measurements are combined with appropriate computational approaches. Such an approach can lead to reliable structural models, as demonstrated by the nano-positioning system recently developed by Michaelis and co-workers [38], [39]. This method uses probabilistic data analysis to combine single-molecule measurements with crystallographic data to determine a three-dimensional probability distribution of a fluorescence label bound to a protein.

In the spin label simulations, the overall performance of the MD simulations to reproduce the experimental distance distributions varies strongly and depends on the sampling at the individual position and therefore on the restrictions imposed by the local environment of the spin label side chain. The MC sampling approach reproduced the experimental distance distributions on average better than the MD simulations, but did also not predict all distance distributions with the same accuracy. Strikingly, the average accuracy of the RLA in predicting the experimental distance distributions is already better than that of MD simulations or MC sampling. Applying an additional rotamer selection based on crystallographic data for spin labeled proteins predicted distance distributions that almost perfectly match the experimental data. This leads to the conclusion that the interaction the rotamer selection is based on, a predicted weak hydrogen-bond between the γ-sulfur of the disulfide link of the spin label side chain and the protein backbone, is relevant for spin labels attached to helical sites not just in crystals but also in (frozen) solutions. In some cases this finding suggests a significant contribution to the encountered deviations when predicting distance distributions from MD simulations or MC sampling: The force fields applied in the simulations seem unable to fully reproduce this kind of side chain-backbone interaction due to the absence of appropriate parameterization of electronic polarizability in the force field. This needs to be tested with more complete sampling and additional examples.

**Figure 8:**
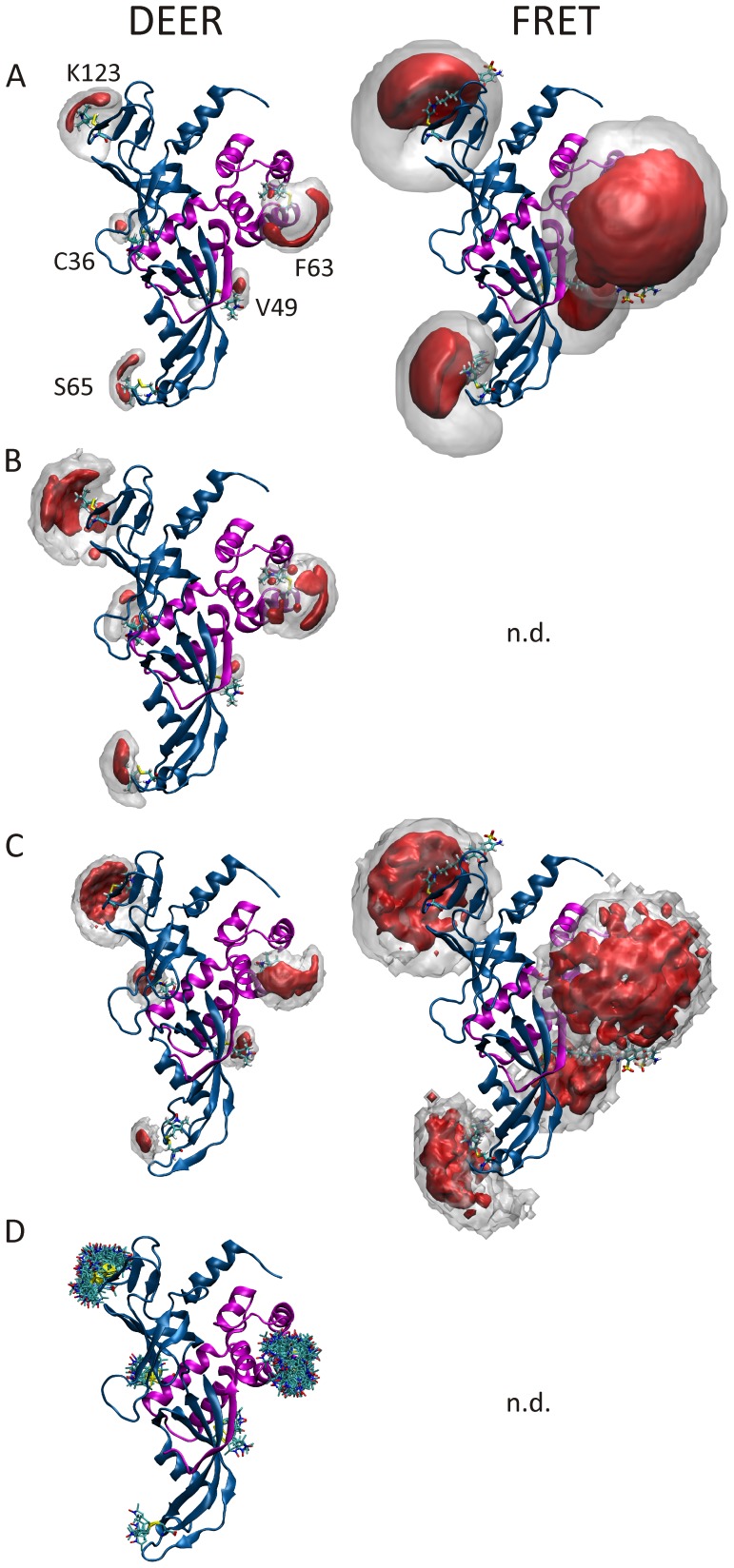
Label orientation probability distributions. deduced from *in vacuo* MD simulations (A), *in aqua* MD simulations (B) and MC sampling (C) in Rpo4/7 (as magenta/blue ribbons). Clouds envelope 99.5% (gray) and 50% (red) of the total probability. Rotamers (D, depicted as sticks) calculated from a given rotamer library [29] span 99.5% of the population. *In aqua* MD simulations have not been performed for the fluorophore labels as they are currently computationally to intensive for standard hardware. A pre-calculated FRET-label rotamer library is currently not available.

It is reasonable to assume that the conformational space and consequently the volume sampled by the label in the respective simulations significantly influence the accuracy of the predicted distance distributions. As can be seen from the respective volume plots (Figs. 5 and 6), but more directly from the spatial probability distributions shown in Figure 8, the methods investigated here show variable performance in covering the full conformational space accessible for the label. The best results are obtained with stochastic sampling, as trapping of the MD simulations in local energy minima appears to be a problem when using this approach. For the *in vacuo* MD performed at significantly elevated temperature (600 K), this trapping seems to be the major issue, as the conformational space sampled turned out to be significantly smaller compared to the other approaches even in spite of the longer trajectory of 100 ns *vs.* 40 ns *in aqua*. For the latter, such sampling difficulties appear to be partially compensated for by the flexibility of the protein backbone. Results similar to the restrained *in vacuo* MD for the volume yet with much narrower distance distributions can be obtained with unrestrained implicit solvent simulations at ambient temperature (data not shown). In contrast, MD simulations in explicit water performed for comparison with position restraints on the backbone atoms showed wider distance distributions while the sampled volume was still limited (data not shown). This suggests a significant influence of the explicit solvent on the conformational sampling of the spin label side chain such that transitions between side chain rotamers are alleviated in the presence of explicit solvent. In the *in aqua* MD simulation and the MC sampling approaches the spin label seems to sample similar volumes, but the different shapes of the probability distributions reveal that the conformational space sampled is different. The RLA is shown to be the best approach in terms of reproducing the experimental distances, and therefore the conformational space occupied by the set of rotamers shown in Figure 8D apparently reflect the “real” situation most accurately, under our assumption that the crystal structure is valid in solution.

### Conclusion

Our study demonstrates that simulations of fluorescent and spin labels can overcome the complications due to their flexibility making them highly applicable for structural investigations. Especially, stochastic simulation approaches enable one to relate FRET distances to structural models. For the simulation of spin label side chains, highly optimized, semi-empirical approaches like the RLA provide efficient means to predict DEER distance data. These results should encourage a more quantitative use of ensemble FRET and DEER to verify or refine structural models of biomolecules and also to combine both techniques as exemplified by Naber et al. [40] and by ourselves [16].

## Materials and Methods

### Recombinant Protein Production

Rpo4 was expressed as a GST-fusion protein and purified using a GST-Trap column (GE Healthcare). Rpo7 was purified by inclusion body isolation [41] and subsequent solubilization in P300 buffer (20 mM Tris/acetate, pH 7.9, 300 mM potassium acetate, 0.1 mM ZnSO_4_, 10 mM magnesium acetate, 10% glycerol) containing 6M urea. In addition to the naturally occurring single cysteine residue in Rpo4 (position 36), single cysteine residues were engineered into RNAP Rpo4 at position G63 (after substitution of the natural cysteine with a serine residue) and Rpo7 at positions V49, S65 or K123 using a splice by overlap extension (SOE) PCR strategy.

### Protein Labeling

For FRET measurements proteins were labeled using the maleimide derivatives of Alexa Fluor 350, Alexa Fluor 488 or Alexa Fluor 594 (Invitrogen), abbreviated as A350, A488 and A594, respectively. Both subunits were labeled under denaturing conditions in the presence of 6 M urea. Rpo4 was precipitated with ammonium sulfate and the resulting pellet was washed three times with 50% ammonium sulfate in 20 mM Tris/HCl pH 7.8, 10 mM EDTA and eventually resuspended in a buffer containing 20 mM Tris/HCl pH 7.8, 10 mM EDTA and 6 M Urea. The protein solution was immediately mixed with a five-fold molar excess of dye over protein and incubated for 2 h at room temperature. Rpo7, purified from inclusion bodies, was labeled in the buffer supplemented with 6 M urea using a five-fold molar excess of dye over protein. The protein-fluorophore mix was incubated for 2 h at room temperature.

For EPR experiments proteins were labeled using MTSSL ((1-oxyl-2,2,5,5-tetramethylpyrroline-3-methyl)methanethiosulfonate spin label). The resulting side chain is denoted R1. Rpo4 and 7 were incubated for 4 h at 4°C with 10 mM DTT in P300 to reduce the cysteine residues. Afterwards, DTT was removed by dialysis using DTT-free P300 and then incubated with 1 mM MTSSL at 4°C over night, which corresponds to a ∼10-fold molar excess. Unbound spin label was removed by 12 hours of dialysis against P300.

Subsequent to the labeling procedure Rpo4 and 7 were dimerized, combining Rpo7 with Rpo4, using a small excess of Rpo7 (molar ratio of 1.5∶1 of Rpo7:Rpo4) and assembled using a denaturation-renaturation approach. Prior to dimerization the fluorophore coupling reaction was stopped by the addition of 1 mM beta-mercaptoethanol. Rpo4 and Rpo7 were combined in 6 M urea and the urea concentration was reduced by step-wise dialysis against buffer solutions containing decreasing amounts of urea using a dialysis frame (Perbio slide-a-lyser, 0.5–3.0 ml). Excess of Rpo7 and misfolded Rpo4/7-complexes were removed by a heat-treatment step (20 min, 65°C) and excess dye and unlabeled Rpo4/7 were removed by anion exchange chromatography (MonoQ, GE Healthcare). The purity and labeling efficiency of the fluorescently labeled proteins was assessed by SDS-PAGE and absorption spectroscopy using an extinction coefficient of 37820 M^−1^ cm^−1^ for the Rpo4/7 heterodimer.

### Fluorescence Measurements

Steady-state ensemble fluorescence measurements were carried out on a FluoroMax-4 (Horiba Jobin Yvon) with a thermostated cuvette holder at 25°C or 65°C in 700 µl or 200 µl quartz cuvettes (Hellma).

Emission spectra of single or double labeled Rpo4/7 complex (50 nM in 50 mM Tris, pH 7.5 and 1 M NaCl) were recorded using an excitation wavelength of 320 nm (A350) or 493 nm (A488). Slits were set to 5 nm.

The measured FRET efficiency is given by:
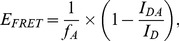
(1)where *I_DA_* is the fluorescence intensity of the donor in the presence of the acceptor and *I_D_* is the fluorescence intensity of the donor in the absence of the acceptor. The apparent efficiencies were corrected by the labeling efficiencies *f_A_* = *c_D_*/*c_P_* with the concentrations of the fluorescence dye, *c_D_*, and of the protein, *c_P_*. Here we used the *f_A_* values for the acceptors, since they have a stronger influence on the resulting *E_FRET_*. The distance *r* between the donor and acceptor can be calculated from the Förster equation:



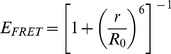
(2)The corresponding Förster radius *R_0_* is given by.
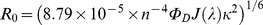
(3)


It is determined by the overlap integral *J*(γ), the donor quantum yield Φ*_D_*, the refractive index *n* and the orientation factor κ^2^ (for a more detailed description see [42]). For the calculation of distances from the measured FRET efficiencies we used approximate values of the Förster radius *R*_0_ as provided by the manufacturer (Invitrogen), which is 50 Å for the A350/488 pair.

Fluorescence anisotropy was measured using an excitation wavelength of 345 nm (A350) or 493 nm (A488) (10 nm slit) and emission wavelength of 444 and 516 nm, respectively. Slits were set to 10 nm for all wavelengths. Parallel and perpendicular emission components were measured in L-Format.

### EPR Spectroscopy

For EPR experiments dimerized Rpo4/7 was concentrated to ∼100 µM, filled into 2 mm inner diameter quartz capillaries and frozen with 20% glycerol for DEER measurements. The spin labeling efficiency determined by continuous-wave EPR- and absorption spectroscopy varied between 70 and 100%.

DEER experiments were performed at X-band frequencies (9.3–9.4 GHz) with a Bruker Elexsys 580 spectrometer equipped with a Bruker Flexline split-ring resonator ER 4118X-MS3 and a continuous flow helium cryostat (CF935; Oxford Instruments) controlled by an Oxford Intelligent Temperature Controller ITC 503S.

All measurements were performed using the four-pulse DEER sequence: π/2(υ_obs_) - τ_1_– π (υ_obs_) – t’ – π (υ_pump_) – (τ_1_+ τ_2_– t’) – π (υ_obs_) - τ_2_– echo [43], [44]. A two-step phase cycling (+ <x>, − <x>) was performed on π/2(υ_obs_). Time t’ is varied, whereas τ_1_ and τ_2_ are kept constant. The dipolar evolution time is given by t = t’ – τ_1_. Data were analyzed only for t >0. The resonator was overcoupled and the pump frequency υ_pump_ was set to the center of the resonator dip (coinciding with the maximum of the nitroxide EPR spectrum) whereas the observer frequency υ_obs_ was 65 MHz higher (low field local maximum of the spectrum). All measurements were performed at a temperature of 50 K with observer pulse lengths of 16 ns for π/2 and 32 ns for π pulses and a pump pulse length of 12 ns. Proton modulation was averaged by adding traces at eight different τ_1_ values, starting at τ_1,0_ = 200 ns and incrementing by Δτ_1_ = 8 ns. For proteins in D_2_O buffer with deuterated glycerol, used for their effect on the phase relaxation, corresponding values were τ_1,0_ = 400 ns and and Δτ_1_ = 56 ns. Data points were collected in 8 ns time steps or, if the absence of fractions in the distance distribution below an appropriate threshold was checked experimentally, in 16 ns time steps. The total measurement time for each sample was 4–24 h.

Analysis of the data was performed with DeerAnalysis 2009 [45].

### Molecular Dynamics Simulations

Prior to simulations two loops and three C-terminal residues missing in the Rpo4/7 crystal structure (PDB 1GO3) [20] were added using VMD [46] with subsequent energy minimization using NAMD [47] with the force field CHARMM27 [48], [49]. Spin labels or fluorescence labels were introduced as a mutation to an artificial side chain in VMD. The spin labeled side chain R1 was generated from the topology described in [50]. The parameters for the side chain containing the fluorophore were kindly provided by Ben Corry [30], while A350 was parameterized by DFT calculations. We obtained an optimized geometry and vibrational frequencies using the functional BP86 [51], [52] and the TZVP [53] basis set with the RI approximation [54] as implemented in ORCA [55]. Partial charges were obtained by fitting them to the molecular electrostatic potential according to Breneman *et al.* [56]. For validation we derived partial charges for other aromatic amino acids and found reasonable agreement with partial charges in CHARMM27. Missing angles and dihedral parameters were all similar to parameters already present in CHARMM27 for the A488 topology and therefore sufficed for the A350 topology. To avoid atomic clashes upon *in silico* labeling, the chromophores were manually directed away from the protein surface. The resulting structures with spin- or fluorescence labels were energy minimized using NAMD.

For *in vacuo* MD simulations we constrained the positions of backbone carbon and nitrogen atoms by the SHAKE algorithm [57]. By coupling the system to a Langevin thermostat [58] with a friction coefficient of 1 ps^−1^ (or 5 ps^−1^ for the 1 ns equilibration period), we maintained the temperature to be 600 K for spin labels according to Beier and Steinhoff [36] and to 2000 K for fluorescence labels according to Wozniak et al. [32]. Additionally, for the fluorescence labeled system electrostatic interactions were disabled to further enhance conformational sampling [31].

For explicit solvent MD simulations, spin labeled Rpo4/7 was immersed in a water box, at least 15 Å larger than Rpo4/7 in any direction, filled with TIP3P water and ∼250 mM sodium and chloride ions, neutralizing the system’s net charge. We applied periodic boundary conditions and used particle mesh Ewald summation [59] to calculate long-range electrostatic interactions. After equilibration of the system at a temperature of 310 K for 200 ps (as described above) the system pressure was equilibrated to an atmospheric level for 300 ps by additional coupling to a barostat according to the Nose-Hoover method [60] with a period and a decay time of 200 ps and 100 ps, and furthermore maintained with 100 ps and 50 ps, respectively.

All MD simulations were carried out with 1 fs time steps, a cutoff for short range electrostatic interactions of 12 Å with a switching function starting at 10 Å, and coordinates were saved in 0.5 ps intervals using NAMD and VMD for calculation, analysis and visualization, respectively. All structure figures were ray-traced in Tachyon [61] with secondary structure assignments by STRIDE [62]. Surfaces were generated by MSMS [63].

### Monte Carlo Sampling

A stochastic conformational search by a Metropolis Monte Carlo (MC) sampling was performed using the AMMP programme package [64] as implemented in the molecular modeling software VEGA ZZ [65]. The force field describing the interatomic energies and partial charges was CHARMM22 [48], [49]. Non-canonical side chains were described in VEGA ZZ according to the atom type description language (ATDL). For the spin label side chain R1 this was described previously [34]. For A350 and A488 the related procedures can be found in the Text S2.

For MC sampling the spin- or fluorescence-labeled structures also used as initial structures for the MD simulations were first subjected to 1,000 conjugate gradient steps of energy minimization in VEGA ZZ to fully relax the structure in the force field CHARMM22. In the subsequent MC sampling, the degrees of freedom for the stochastic jumps were limited to the flexible dihedral angles, five angles for the spin labels and eleven angles for the fluorescence labels with a minimum total dihedral RMSD per jump of 20° or 50°, respectively. In this putative new state, the protein was energy minimized (100 conjugate gradient steps) before state acceptance was assessed by the Metropolis criterion [66] using the CHARMM22 energy at a temperature of 300 K in a dielectric continuum with ε = 80. For each label we generated a trajectory of *N* = 50,000 MC sampling steps independent of the conformation of other labels and analyzed label pairs from the set non-synchronously, i.e. using a sliding window, where for each frame of the first label distances to all frames of the second label were calculated and subsequently, we combined all *N^2^* distances in a histogram preserving the approximately Boltzmann distributed energies in the simulated canonical ensembles.

### Rotamer Library Analysis

In the rotamer library analysis (RLA) the canonical ensemble of spin label side chain conformations is modeled by a discrete set of 210 precalculated rotamers [29]. From the RLA a conformational distribution of R1 at any chosen position in the otherwise fixed protein structure can be determined as described in detail in [29]. In brief, the superposition of the R1 backbone atoms onto the protein backbone at the respective position provides the orientation of R1 with respect to the protein structure and allows for the calculation of a resulting energy for the R1-protein interaction from the Lennard-Jones potential using the MD force field CHARMM27 [67]. Subsequent Boltzmann weighting and normalization by the partition function yields a probability for each rotamer which is then multiplied by the probability of R1 to exhibit each conformation. This results in the final rotamer probability distribution at the site of interest. Between two such probability distributions at two positions in the protein, a distance distribution is calculated as the histogram of all pairwise interspin distances weighted by the product of their respective probabilities. The RLA is performed with the freely available software package MMM (Multiscale Modeling of Macromolecules, version 2010) [29].

## Supporting Information

Figure S1 **Structural alignment of the** ***M. jannaschii*** **,** ***Sulfolobus solfataricus*** **and** ***Sulfolobus shibatae*** **Rpo4/7 complexes.** The structure alignment of RPB7 and RPB4 shows the clear conservation of the structures of both subunits between the three archaea compared: *Methanocaldococcus jannaschii* (blue, PDB 1GO3), *Sulfolobus solfataricus* (red, PDB 2PMZ) and *Sulfolobus shibatae* (green, PDB 2WAQ).
(TIF)

Figure S2 **Additional** ***in vacuo*** MD and MC trajectories and volume plots for for the FRET pairs Rpo4^G63C*A488^/Rpo7^S65C*A350^ (A: ***in vacuo*** MD, C: MC) and Rpo4^G63C*A488^/Rpo7^K123C*A350^ (B: ***in vacuo*** **MD, D: MC) with the distance trajectories in the left column and the respective volume plots in the middle and right columns.**
(TIF)

Figure S3 **Comparison of MC samplings with 50000 and 100000 steps.** (A) Distance distribution obtained from MC samplings with 50000 steps (red) and 100000 steps (black). (B) Distance trajectory (left column) and volume plots (middle and right columns) from the MC samplings with 100000 steps. (C) Distance trajectory (left column) and volume plots (middle and right columns) from the MC sampling with 50000 steps.
(TIF)

Figure S4 Spatial probability distributions for fluorescence labels in Rpo4^G63C*A488^ and Rpo7^V49C*A350^ deduced from ***in vacuo*** **MD (blue) simulations and MC sampling (red).** Clouds envelope 99.5% (gray) and 50% (blue/red) of the total probability. A shift of the distributions (MC *vs.* MD) is observed for Rpo4/Rpo7^V49C*A350^.
(TIF)

Figure S5 **Additional in vacuo MD and MC trajectories and volume plots for spin labels in Rpo4^G63R1^/Rpo7^xR1^.** Distance trajectories (left column) and volume plots (middle and right columns) for *in vacuo* MD simulations with (A) Rpo4^G63R1^/Rpo7^S65R1^ and (B) Rpo4^G63R1^/Rpo7^K123R1^, and MC samplings (50000 steps) with (C) Rpo4^G63R1^/Rpo7^S65R1^ and (D) Rpo4^G63R1^/Rpo7^K123R1^.
(TIF)

Figure S6 **Additional** ***in vacuo*** **MD and MC trajectories and volume plots for spin labels in Rpo4^C36R1^/Rpo7^xR1^.** Distance trajectories (left column) and volume plots (middle and right columns) for *in vacuo* MD simulations with (A) Rpo4^C36R1^/Rpo7^S65R1^ and (B) Rpo4^C36R1^/Rpo7^K123R1^, and MC samplings (50000 steps) with (C) Rpo4^C36R1^/Rpo7^S65R1^ and (D) Rpo4^C36R1^/Rpo7^K123R1^.
(TIF)

Figure S7 **Additional** ***in aqua*** **MD trajectories and volume plots for spin labels in Rpo4^G63R1^/Rpo7^xR1^ and Rpo4^C36R1^/Rpo7^xR1^.** Distance trajectories (left column) and volume plots (middle and right columns) for (A) Rpo4^G63R1^/Rpo7^S65R1^, (B) Rpo4^G63R1^/Rpo7^K123R1^, (C) Rpo4^C36R1^/Rpo7^S65R1^ and (D) Rpo4^C36R1^/Rpo7^K123R1^.
(TIF)

Figure S8 **X1/X2 rotamer selection in RLA.** (A) MTS-labeled side chain R1, the first two dihedral angles, X1 and X2, are indicated by arrows. (B) Variety of states in the MTSSL 210-rotamer library. All 213 MTSSL-rotamers span 9 groups in the plane of the R1 dihedral angles X1/X2 [69]. In a previous study [68] MTSSL was found to exhibit only three rotamers {X1,X2} in protein crystals at α-helical sites for both cryogenic and ambient temperatures. Of those three, only the rotamers {m,m} and {t,p} are highly populated possibly due to the stabilizing formation of a weak intra-MTSSL hydrogen bond: Cα -Hα ⋅⋅⋅Sδ. Here, for the rotamer distributions at the α-helical positions Rpo4^G63R1^ and Rpo4^C36R1^, selection of only {m,m} and {t,p} within the dihedral angle distributions leads to altered distance distributions which fit the experimental data best.
(TIF)

Text S1 **Tikhonov regularization and DEER data analyses.** A brief description of the theoretical background and procedure of DEER data analysis by Tikhonov regularization, and detailed analyses of the DEER data presented in this paper.
(DOC)

Text S2 **Atom type definitions used for MC sampling.** Atom types added to the VegaZZ template CHARMM22_PRO for MC sampling of the fluorophores.
(DOC)

## References

[1] Agafonov RV, Negrashov IV, Tkachev YV, Blakely SE, Titus MA (2009). Structural dynamics of the myosin relay helix by time-resolved EPR and FRET.. Proc Natl Acad Sci U S A.

[2] Edidin M (2003). Fluorescence resonance energy transfer: techniques for measuring molecular conformation and molecular proximity.. Curr Prot Immunol Chapter 18: Unit 18.10.

[3] Schiemann O, Prisner TF (2007). Long-range distance determinations in biomacromolecules by EPR spectroscopy.. Q Rev Biophys.

[4] Bordignon E, Steinhoff HJ (2007). Membrane protein structure and dynamics studied by site-directed spin labeling ESR. In ESR Spectroscopy in Membrane Biophysics, Hemminga, M. A., Berliner, L. J., Eds.; New York: Springer Science and Business Media.. Pp.

[5] Förster T (1959). Transfer mechanisms of electronic excitation.. Discuss Faraday Soc.

[6] Grohmann D, Nagy J, Chakraborty A, Klose D, Fielden D (2011). The Initiation Factor TFE and the Elongation Factor Spt4/5 Compete for the RNAP Clamp during Transcription Initiation and Elongation.. Mol Cell.

[7] Ha T, Enderle T, Ogletree DF, Chemla DS, Selvin PR (1996). Probing the interaction between two single molecules: fluorescence resonance energy transfer between a single donor and a single acceptor.. Proc Natl Acad Sci U S A.

[8] Sisamakis E, Valeri A, Kalinin S, Rothwell PJ, Seidel CAM (2010). Accurate Single-Molecule FRET Studies Using Multiparameter Fluorescence Detection.. Method Enzymol.

[9] Kay CWM, El Mkami H, Cammack R, Evans RW (2007). Pulsed ELDOR Determination of the Intramolecular Distance between the Metal Binding Sites in Dicupric Human Serum Transferrin and Lactoferrin.. J Am Chem Soc.

[10] Kay CWM, Elsässer C, Bittl R, Farrell SR, Thorpe C (2006). Determination of the Distance between the Two Neutral Flavin Radicals in Augmenter of Liver Regeneration by Pulsed ELDOR.. J Am Chem Soc.

[11] Altenbach C, Flitsch SL, Khorana HG, Hubbell WL (1989). Structural studies on transmembrane proteins. 2. Spin labelling of bacteriorhodopsin mutants at unique cysteines.. Biochemistry.

[12] Klare JP, Steinhoff HJ (2009). Spin Labling EPR.. Photosynth Res.

[13] Liang B, Bushweller BH, Tamm LK (2006). Site-directed Parallel Spin-Labeling and Paramagnetic Relaxation Enhancement in Structure Determination of Membrane Proteins by Solution NMR Spectroscopy.. J Am Chem Soc.

[14] Werner F, Grohmann D (2011). Evolution of multisubunit RNA polymerases in the three domains of life.. Nat Rev Microbiol.

[15] Hirtreiter A, Damsma GE, Cheung ACM, Klose D, Grohmann D (2010). Spt4/5 stimulates transcription elongation through the RNA polymerase clamp coiled-coil motif.. Nucl Acids Res.

[16] Grohmann D, Klose D, Klare JP, Kay CWM, Steinhoff HJ (2010). RNA-Binding to Archaeal RNA Polymerase Subunits F/E: A DEER and FRET Study.. J Am Chem Soc.

[17] Meka H, Werner F, Cordell SC, Onesti S, Brick P (2005). Crystal structure and RNA binding of the Rpb4/Rpb7 subunits of human RNA polymerase II.. Nucl Acids Res.

[18] Snare MJ, Treloar FE, Ghiggino KP, Thistlethwaite PJ (1982). The photophysics of rhodamine B. J Photochem.

[19] Shah JJ, Gaitan M, Geist J (2009). Generalized Temperature Measurement Equations for Rhodamine B Dye Solution and Its Application to Microfluidics.. Anal Chem.

[20] Todone F, Brick P, Werner F, Weinzierl ROJ, Onesti S (2001). Structure of an Archaeal Homolog of the Eukaryotic RNA Polymerase II RPB4/RPB7 Complex.. Mol Cell.

[21] Dietrich A, Buschmann V, Müller C, Sauer M (2002). Fluorescence resonance energy transfer (FRET) and competing processes in donor-acceptor substituted DNA strands: a comparative study of ensemble and single-molecule data.. Rev Mol Biotechnol.

[22] Marras SAE, Kramer FR, Tyagi S (2002). Efficiencies of fluorescence resonance energy transfer and contact-mediated quenching in oligonucleotide probes.. Nucl Acids Res.

[23] Förster T (1967). Mechanism of energy transfer.. In Comprehensive Biochemistry, Bioenergetics, Elsevier: Amsterdam.

[24] Wu P, Brand L (1992). Orientation factor in steady-state and time-resolved resonance energy transfer measurements.. Biochem.

[25] dos Remedios CG, Moens PDJ (1995). Fluorescence Resonance Energy Transfer Spectroscopy Is a Reliable "Ruler" for Measuring Structural Changes in Proteins : Dispelling the Problem of the Unknown Orientation Factor.. J Struct Biol.

[26] Clegg RM (1992). Fluorescence resonance energy transfer and nucleic acids.. Method Enzymol.

[27] Majumdar ZK, Hickerson R, Noller HF, Clegg RM (2005). Measurements of Internal Distance Changes of the 30 S Ribosome Using FRET with Multiple Donor-Acceptor Pairs: Quantitative Spectroscopic Methods.. J Mol Biol.

[28] Alexander N, Al-Mestarihi A, Bortolus M, Mchaourab HS, Meiler S (2008). De Novo High-Resolution Structure Determination from Sparse Spin-Labeling EPR Data.. Structure.

[29] Polyhach Y, Bordignon E, Jeschke G (2011). Rotamer libraries of spin labelled cysteines for protein studies.. Phys Chem Chem Phys.

[30] Corry B, Jayatilaka D (2008). Simulation of Structure, Orientation, and Energy Transfer between AlexaFluor Molecules Attached to MscL.. Biophys J.

[31] Schröder GF, Alexiev U, Grubmüller H (2005). Simulation of Fluorescence Anisotropy Experiments: Probing Protein Dynamics.. Biophys J.

[32] Wozniak AK, Schröder GF, Grubmüller H, Seidel CAM, Oesterhelt F (2008). Single-molecule FRET measures bends and kinks in DNA.. Proc Natl Acad Sci U S A.

[33] Steinhoff HJ (2004). Inter- and intra-molecular distances determined by EPR spectroscopy and site-directed spin labeling reveal protein-protein and protein-oligonucleotide interaction.. Biol Chem.

[34] Boehme S, Padmavathi PVL, Holterhues J, Ouchni F, Klare JP (2009). Topology of the amphipathic helices of the colicin A pore-forming domain in E. coli lipid membranes studied by pulse EPR.. Phys Chem Chem Phys.

[35] Jeschke G, Polyhach Y (2007). Distance measurements on spin-labelled biomacromolecules by pulsed electron paramagnetic resonance.. Phys Chem Chem Phys.

[36] Beier C, Steinhoff HJ (2006). A Structure-Based Simulation Approach for Electron Paramagnetic Resonance Spectra Using Molecular and Stochastic Dynamics Simulations.. Biophys J.

[37] Fleissner MR, Cascio D, Hubbell WL (2009). Structural origin of weakly ordered nitroxide motion in spin-labeled proteins.. Protein Sci.

[38] Muschielok A, Andrecka J, Jawhari A, Bruckner F, Cramer P (2008). A nano-positioning system for macromolecular structural analysis.. Nat Methods.

[39] Andrecka J, Treutlein B, Arcusa MAI, Muschielok A, Lewis R (2009). Nano positioning system reveals the course of upstream and nontemplate DNA within the RNA polymerase II elongation complex.. Nucl Acids Res.

[40] Naber N, Malnasi-Csizmadia A, Purcell TJ, Cooke R, Pate E (2010). Combining EPR with Fluorescence Spectroscopy to Monitor Conformational Changes at the Myosin Nucleotide Pocket.. J Mol Biol.

[41] Werner F, Weinzierl ROJ (2002). A Recombinant RNA Polymerase II-like Enzyme Capable of Promoter-Specific Transcription.. Mol Cell.

[42] Lakowicz JR (2006). New York: Springer..

[43] Martin RE, Pannier M, Diederich F, Gramlich V, Hubrich M (1998). Determination of End-to-End Distances in a Series of TEMPO Diradicals of up to 2.8 nm Length with a New Four-Pulse Double Electron Electron Resonance Experiment.. Angew Chem Int Ed.

[44] Pannier M, Veit S, Godt A, Jeschke G, Spiess HW (2000). Dead-Time Free Measurement of Dipole-Dipole Interactions between Electron Spins.. J Magn Res.

[45] Jeschke G, Chechik V, Ionita P, Godt A, Zimmermann H (2006). DeerAnalysis2006 - a comprehensive software package for analyzing pulsed ELDOR data.. Appl Magn Reson.

[46] Humphrey W, Dalke A, Schulten K (1996). VMD: Visual molecular dynamics.. J Mol Graph.

[47] Phillips JC, Braun R, Wang W, Gumbart J, Tajkhorshid E (2005). Scalable molecular dynamics with NAMD.. J Comput Chem.

[48] Brooks BR, Bruccoleri RE, Olafson BD, States DJ, Swaminathan S (1983). CHARMM: A program for macromolecular energy, minimization, and dynamics calculations.. J Comput Chem.

[49] Brooks BR, Brooks CL, Mackerell AD, Nilsson L, Petrella RJ (2009). CHARMM: The biomolecular simulation program.. J Comput Chem.

[50] Fajer PG, Brown L, Song L (2007). Practical Pulsed Dipolar ESR (DEER). In ESR Spectroscopy in Membrane Biophysics, Hemminga, M. A., Berliner, L. J., Eds.; New York: Springer Science and Business Media.. Pp.

[51] Perdew JP (1986). Density-functional approximation for the correlation energy of the inhomogeneous electron gas.. Phys Rev B.

[52] Becke AD (1988). Density-functional exchange-energy approximation with correct asymptotic behavior.. Phys Rev A.

[53] Schäfer A, Huber C, Ahlrichs R (1994). Fully optimized contracted Gaussian basis sets of triple zeta valence quality for atoms Li to Kr.. J Chem Phys.

[54] Vahtras O, Almlöf J, Feyereisen MW (1993). Integral approximations for LCAO-SCF calculations.. Chem Phys Lett.

[55] ORCA (2007). an ab initio, Density Functional und Semiempirical Program Package 2.6.0, version 2.6.0..

[56] Breneman CM, Wiberg KB (1990). Determining atom-centered monopoles from molecular electrostatic potentials. The need for high sampling density in formamide conformational analysis.. J Comput Chem.

[57] Van Gunsteren WF, Berendsen HJC (1982). Algorithms for brownian dynamics.. Mol Phys.

[58] Brünger A, Brooks CL, Karplus M (1984). Stochastic boundary conditions for molecular dynamics simulations of ST2 water.. Chem Phys Lett.

[59] Darden T, York D, Pedersen L (1993). Particle mesh Ewald: An N · log(N) method for Ewald sums in large systems.. J Chem Phys.

[60] Martyna GJ, Tobias DJ, Klein ML (1994). Constant pressure molecular dynamics algorithms.. J Chem Phys.

[61] Stone JE (1998). An Efficient Library for Parallel Ray Tracing and Animation.. In: Proceedings of the 1995 Intel Supercomputer Users Group Conference; University of Missouri-Rolla, Missouri, United States..

[62] Frishman D, Argos P (1995). Knowledge-based protein secondary structure assignment.. Proteins.

[63] Sanner MF, Olson AJ, Spehner JC (1996). Reduced surface: An efficient way to compute molecular surfaces.. Biopolymers.

[64] Harrison RW (1993). Stiffness and energy conservation in molecular dynamics: An improved integrator.. J Comput Chem.

[65] Pedretti A, Villa L, Vistoli G (2002). VEGA: a versatile program to convert, handle and visualize molecular structure on Windows-based PCs.. J Mol Graph Model.

[66] Metropolis N, Rosenbluth AW, Rosenbluth MN, Teller AH, Teller E (1953). Equation of State Calculations by Fast Computing Machines.. J Chem Phys.

[67] Mackerell AD, Feig M, Brooks CL (2004). Extending the treatment of backbone energetics in protein force fields: Limitations of gas-phase quantum mechanics in reproducing protein conformational distributions in molecular dynamics simulations.. J Comput Chem.

[68] Fleissner M, Hubbell WL (2009). Protein Sci..

[69] Polyhach Y (2011). Phys. Chem. Chem. Phys..

