# Supplementary figures and images for: Simulation vs. Reality: A Comparison of In Silico Distance Predictions with DEER and FRET Measurements
Source: PLoS One. 2012 Jun 25;7(6):e39492. doi: 10.1371/journal.pone.0039492 (PMC3382601; doi:10.1371/journal.pone.0039492)

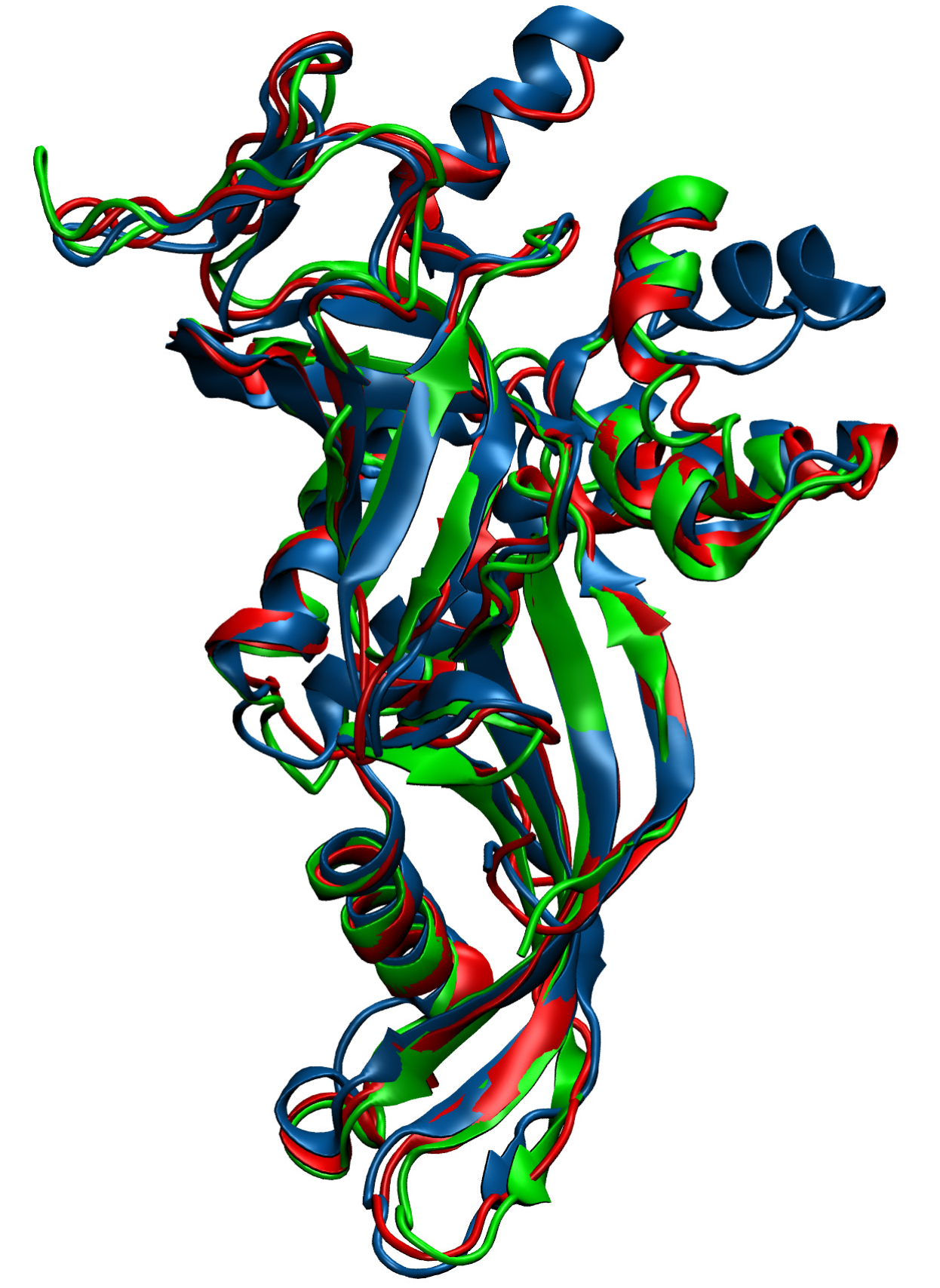

Supplement: Figure S1 — Structural alignment of the M. jannaschii , Sulfolobus solfataricus and Sulfolobus shibatae Rpo4/7 complexes. The structure alignment of RPB7 and RPB4 shows the clear conservation of the structures of both subunits between the three archaea compared: Methanocaldococcus jannaschii (blue, PDB 1GO3), Sulfolobus solfataricus (red, PDB 2PMZ) and Sulfolobus shibatae (green, PDB 2WAQ). (TIF) [file pone.0039492.s001.tif]

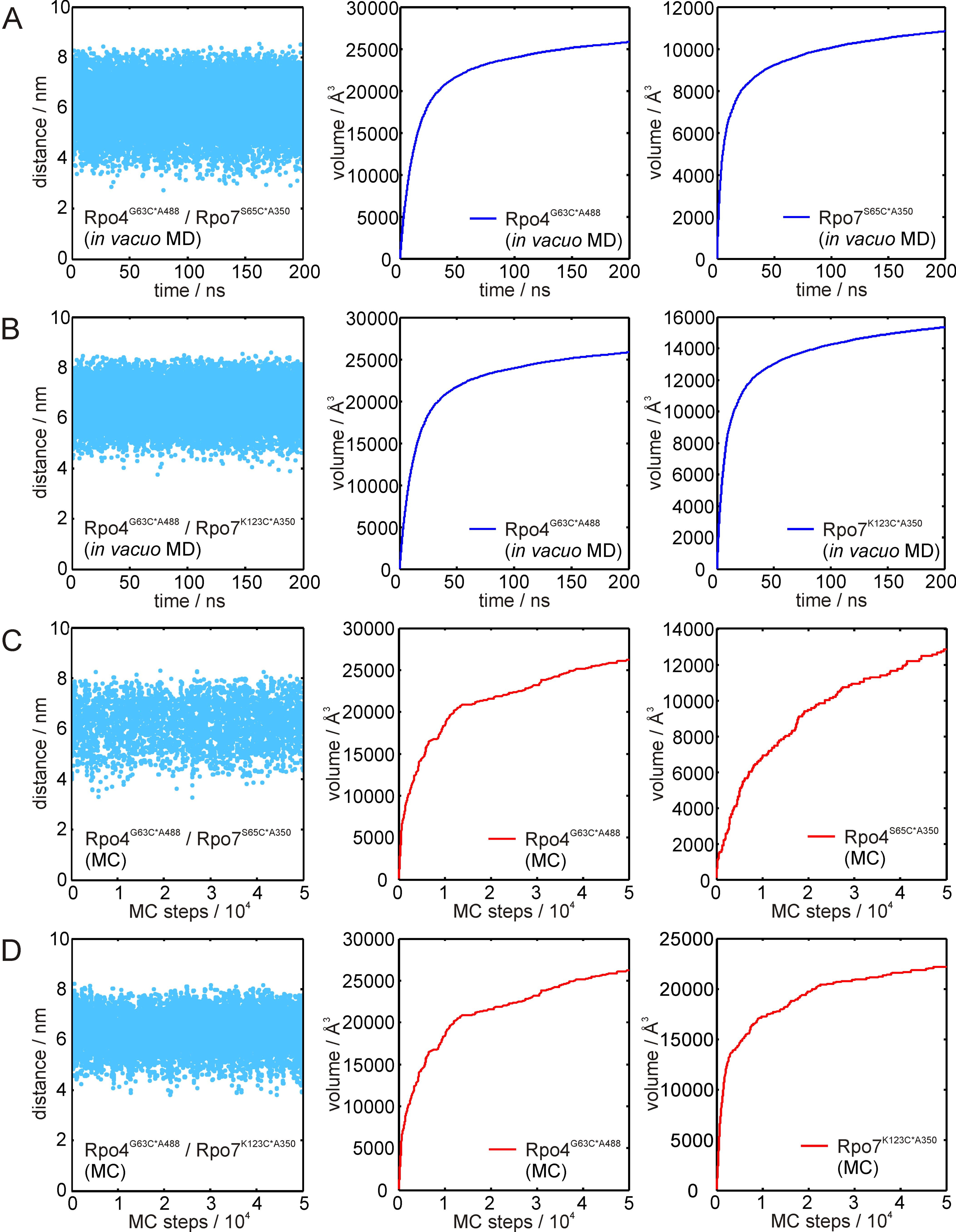

Supplement: Figure S2 — Additional in vacuo MD and MC trajectories and volume plots for for the FRET pairs Rpo4G63C*A488/Rpo7S65C*A350 (A: in vacuo MD, C: MC) and Rpo4G63C*A488/Rpo7K123C*A350 (B: in vacuo MD, D: MC) with the distance trajectories in the left column and the respective volume plots in the middle and right columns. (TIF) [file pone.0039492.s002.tif]

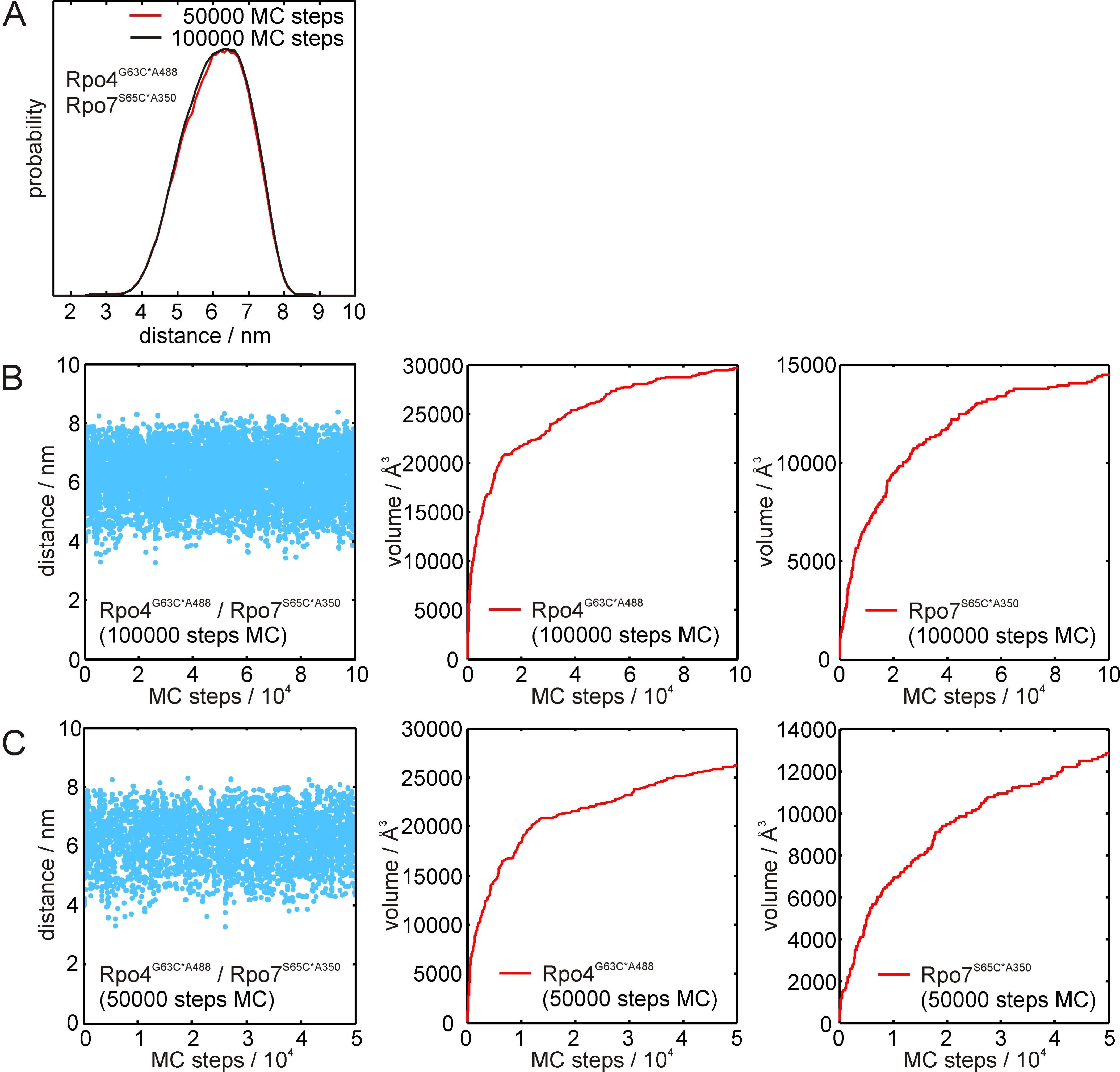

Supplement: Figure S3 — Comparison of MC samplings with 50000 and 100000 steps. (A) Distance distribution obtained from MC samplings with 50000 steps (red) and 100000 steps (black). (B) Distance trajectory (left column) and volume plots (middle and right columns) from the MC samplings with 100000 steps. (C) Distance trajectory (left column) and volume plots (middle and right columns) from the MC sampling with 50000 steps. (TIF) [file pone.0039492.s003.tif]

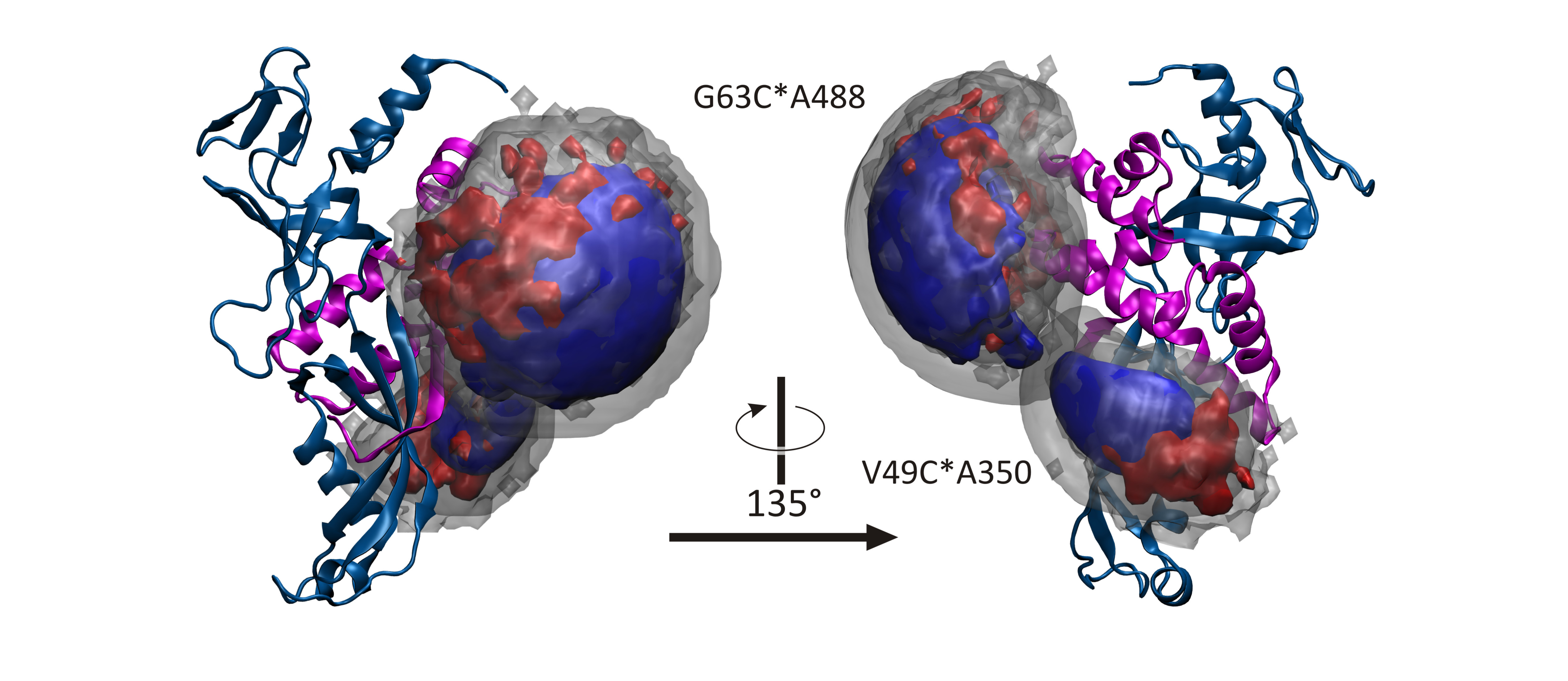

Supplement: Figure S4 — Spatial probability distributions for fluorescence labels in Rpo4G63C*A488 and Rpo7V49C*A350 deduced from in vacuo MD (blue) simulations and MC sampling (red). Clouds envelope 99.5% (gray) and 50% (blue/red) of the total probability. A shift of the distributions (MC vs. MD) is observed for Rpo4/Rpo7V49C*A350. (TIF) [file pone.0039492.s004.tif]

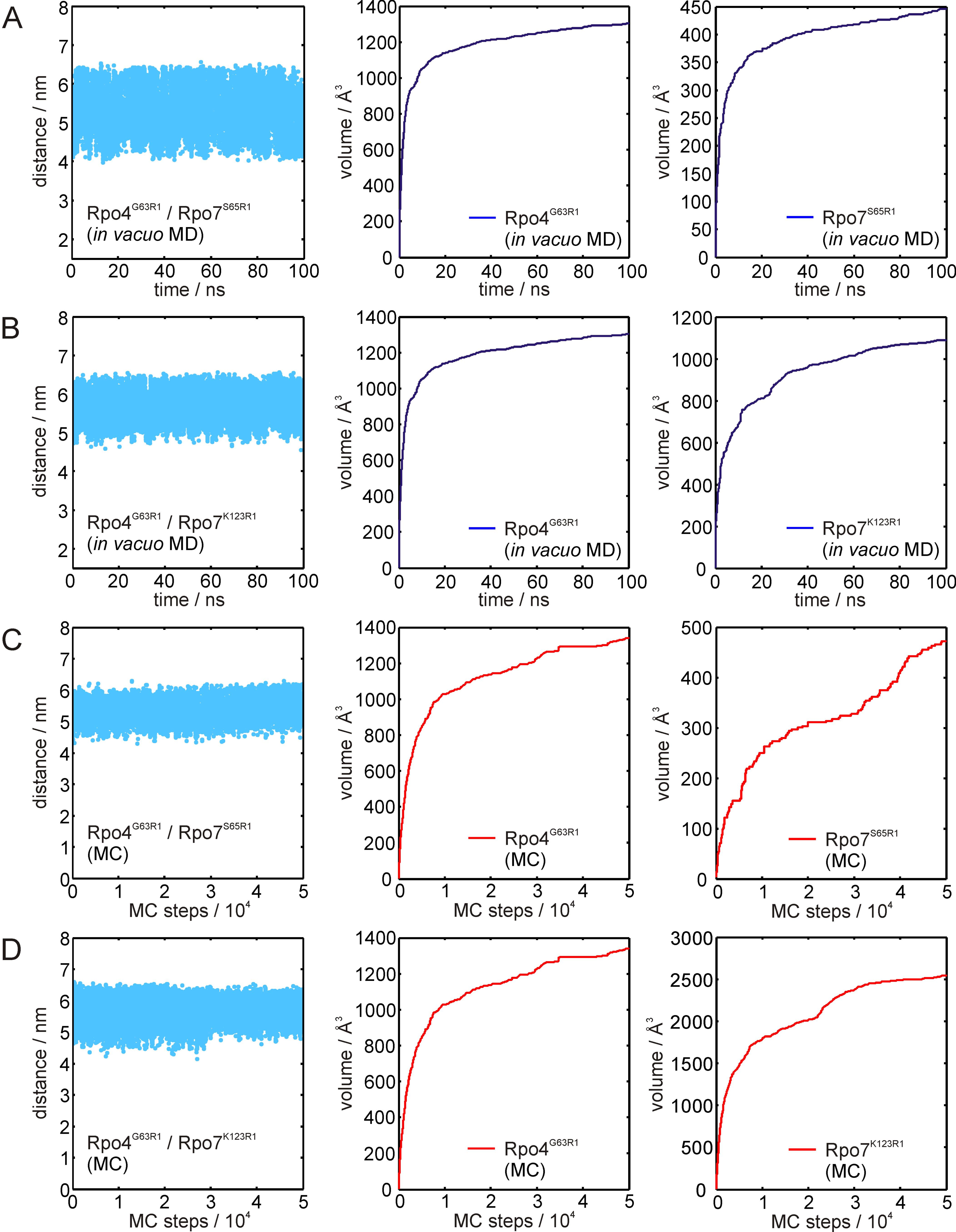

Supplement: Figure S5 — Additional in vacuo MD and MC trajectories and volume plots for spin labels in Rpo4G63R1/Rpo7xR1. Distance trajectories (left column) and volume plots (middle and right columns) for in vacuo MD simulations with (A) Rpo4G63R1/Rpo7S65R1 and (B) Rpo4G63R1/Rpo7K123R1, and MC samplings (50000 steps) with (C) Rpo4G63R1/Rpo7S65R1 and (D) Rpo4G63R1/Rpo7K123R1. (TIF) [file pone.0039492.s005.tif]

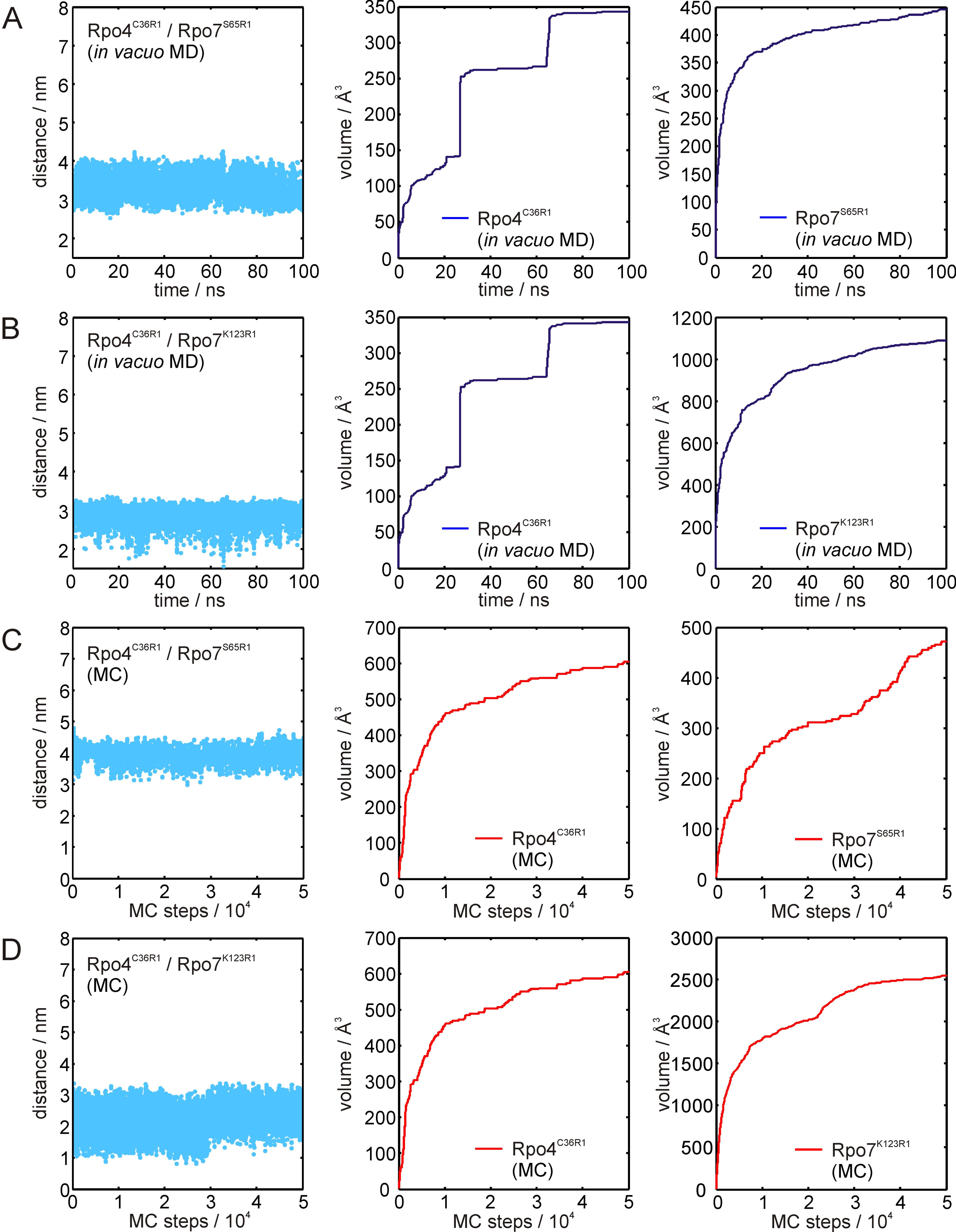

Supplement: Figure S6 — Additional in vacuo MD and MC trajectories and volume plots for spin labels in Rpo4C36R1/Rpo7xR1. Distance trajectories (left column) and volume plots (middle and right columns) for in vacuo MD simulations with (A) Rpo4C36R1/Rpo7S65R1 and (B) Rpo4C36R1/Rpo7K123R1, and MC samplings (50000 steps) with (C) Rpo4C36R1/Rpo7S65R1 and (D) Rpo4C36R1/Rpo7K123R1. (TIF) [file pone.0039492.s006.tif]

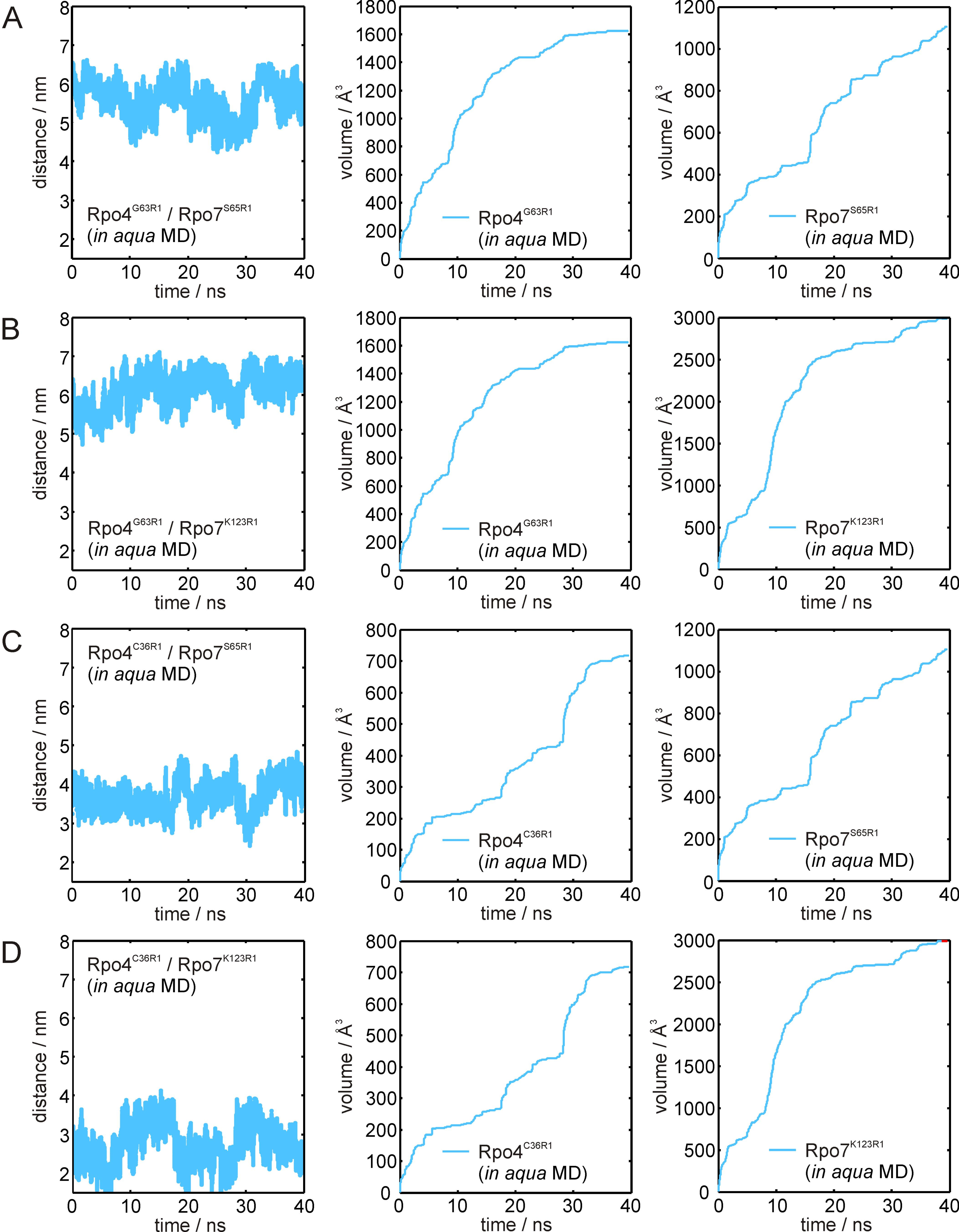

Supplement: Figure S7 — Additional in aqua MD trajectories and volume plots for spin labels in Rpo4G63R1/Rpo7xR1 and Rpo4C36R1/Rpo7xR1. Distance trajectories (left column) and volume plots (middle and right columns) for (A) Rpo4G63R1/Rpo7S65R1, (B) Rpo4G63R1/Rpo7K123R1, (C) Rpo4C36R1/Rpo7S65R1 and (D) Rpo4C36R1/Rpo7K123R1. (TIF) [file pone.0039492.s007.tif]

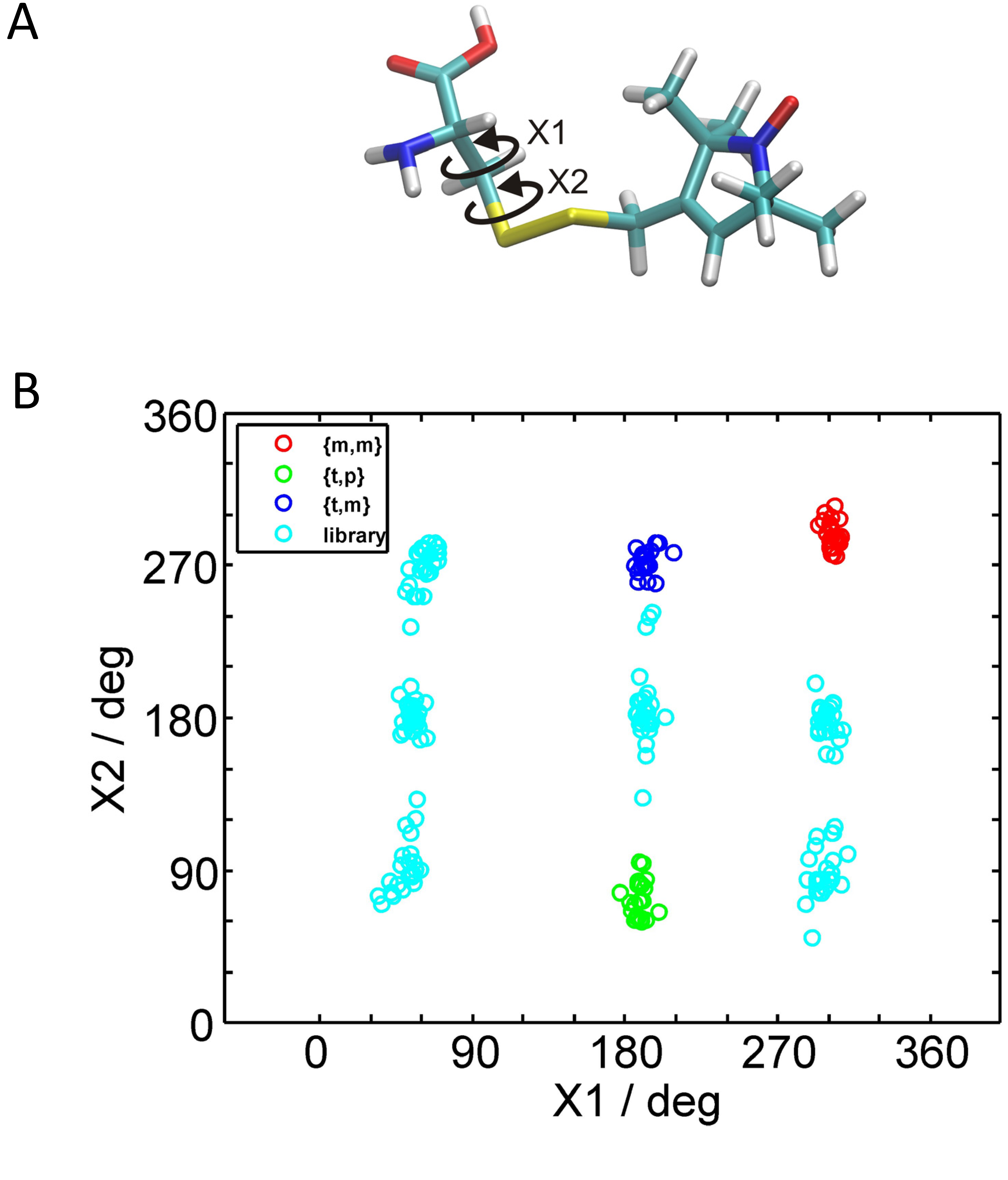

Supplement: Figure S8 — X1/X2 rotamer selection in RLA. (A) MTS-labeled side chain R1, the first two dihedral angles, X1 and X2, are indicated by arrows. (B) Variety of states in the MTSSL 210-rotamer library. All 213 MTSSL-rotamers span 9 groups in the plane of the R1 dihedral angles X1/X2 [69]. In a previous study [68] MTSSL was found to exhibit only three rotamers {X1,X2} in protein crystals at α-helical sites for both cryogenic and ambient temperatures. Of those three, only the rotamers {m,m} and {t,p} are highly populated possibly due to the stabilizing formation of a weak intra-MTSSL hydrogen bond: Cα -Hα ⋅⋅⋅Sδ. Here, for the rotamer distributions at the α-helical positions Rpo4G63R1 and Rpo4C36R1, selection of only {m,m} and {t,p} within the dihedral angle distributions leads to altered distance distributions which fit the experimental data best. (TIF) [file pone.0039492.s008.tif]
