# Supplementary material for: Simulation vs. Reality: A Comparison of In Silico Distance Predictions with DEER and FRET Measurements
Source: PLoS One. 2012 Jun 25;7(6):e39492. doi: 10.1371/journal.pone.0039492 (PMC3382601; doi:10.1371/journal.pone.0039492)

**Supplementary Text S1: Tikhonov Regularization and DEER data analyses**

Calculation of a distance distribution *P(r)* from experimental DEER data requires the following steps: The experimental raw data (or dipolar evolution function) *S*(*t*) is a product of the so called form factor *F(t)* and a background *B(t)* from the homogeneous distribution of surrounding molecules carrying dipolarly coupled spins. Therefore, the form factor is obtained by division with the background fitted to the raw data after the decay of initial dipolar oscillations.

From any given distance distribution the calculation of the according form factor *F(t)* is straight forward [1], yet the inverse problem of calculating a distance distribution *P(r)* from *F(t)* is ill-posed, i.e. not well defined, because of its degeneracy: different *P(r)* can give rise to the same *F(t)*.

The adequate mathematical tool to solve such an ill-posed problem is to stabilize the solution by adding a second measure (here the curvature/smoothness of *P(r)*) in addition to the quality of the fit to *F(t)*. Furthermore the problem is solved under the side constraint *P(r)* > 0 for all distances r.

Finally, since we now have two measures for a successful solution, namely the fit quality and the smoothness of *P(r)*, we need to find a proper weighting factor between these two measures; this is the so-called regularization parameter α. In summary, for one regularization parameter α, *P(r)* is calculated by minimizing the objective function:

$G_{\alpha}(P)=\left\|S(t)-D(t)\right\|^{2}+\alpha\cdot\left\|\frac{d^{2}}{dr^{2}}P(r)\right\|_{\alpha}^{2}$,

with the means square deviation $\rho(\alpha)=\left\|S(t)-D(t)\right\|^{2}$, where *D*(*t*) is the simulated dipolar evolution function, and the “smoothness” of the distance distribution $\eta(a)=\left\|\frac{d^{2}}{dr^{2}}P(r)\right\|_{\alpha}^{2}$.

Determination of a proper α is part of finding a solution for the calculation of *P(r)*. The mathematical possibility which is least susceptible to experimental noise is called L curve criterion. For a set of solutions calculated with different α, the plot of the log(smoothness η) over the log(deviation ρ) of the fit from *F(t)* typically has an L-shaped form with the ideal α lying at the kink [2]. Graphs according to this criterion are used here to determine adequate α values as implemented in DeerAnalysis2010 [3]. Analyses of the data sets from this study are shown on the next pages (from top, left to bottom, right: *S*(*t*) and background correction, resulting *F*(*t*) with fit from Tikhonov regularization, dipolar spectrum (Pake Pattern = Fourier transform of *F*(*t*)), L-curve, and resulting distance distribution(s) .

References:

1. Pannier, M. et al., J. Magn. Reson. 142 (2000) 331-340.

2. Jeschke, G. et al., Appl. Magn. Reson. 30 (2006) 473-498.

3. Chiang, Y. W., et al., J. Magn. Reson. 172 (2005) 279-295.


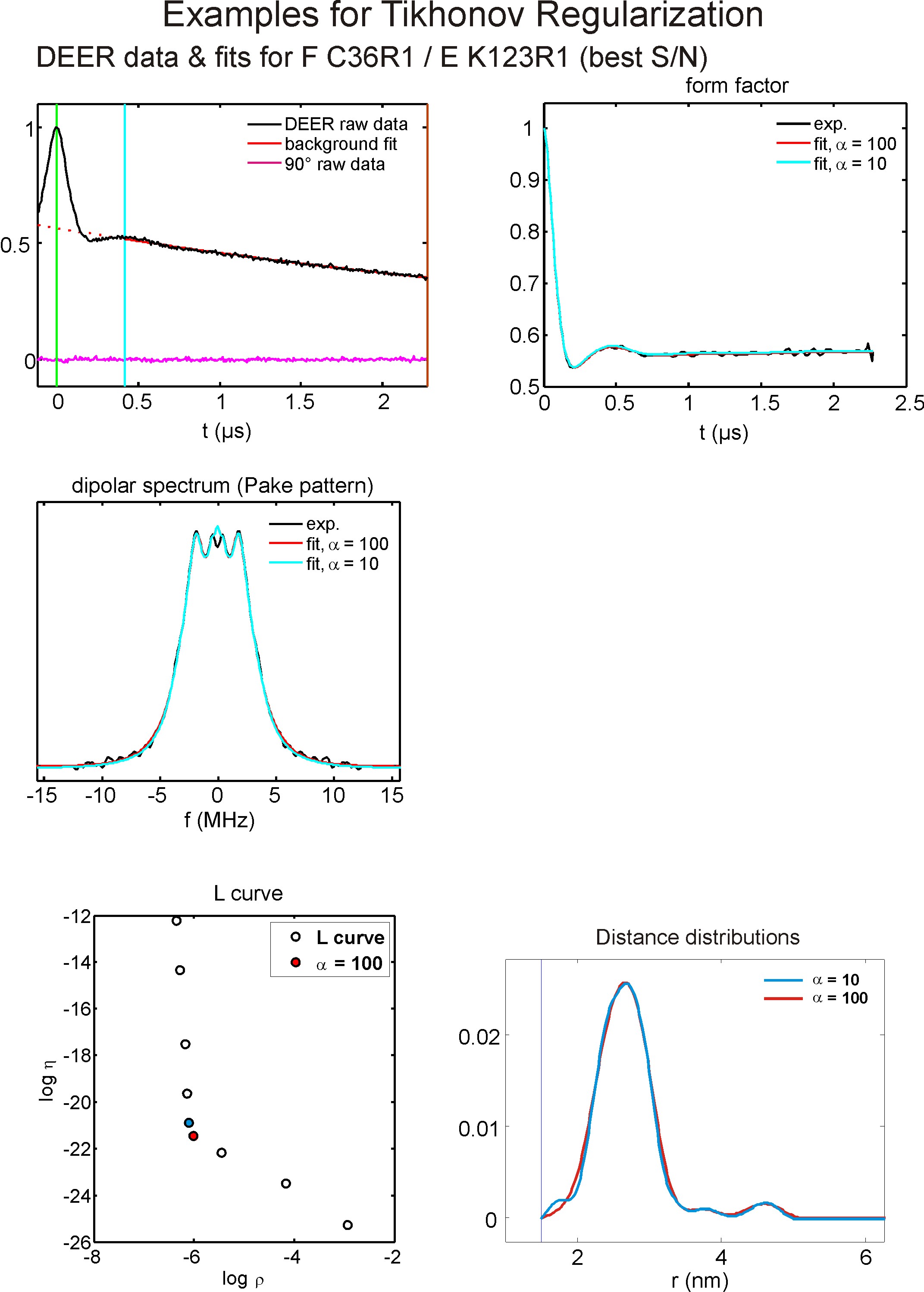


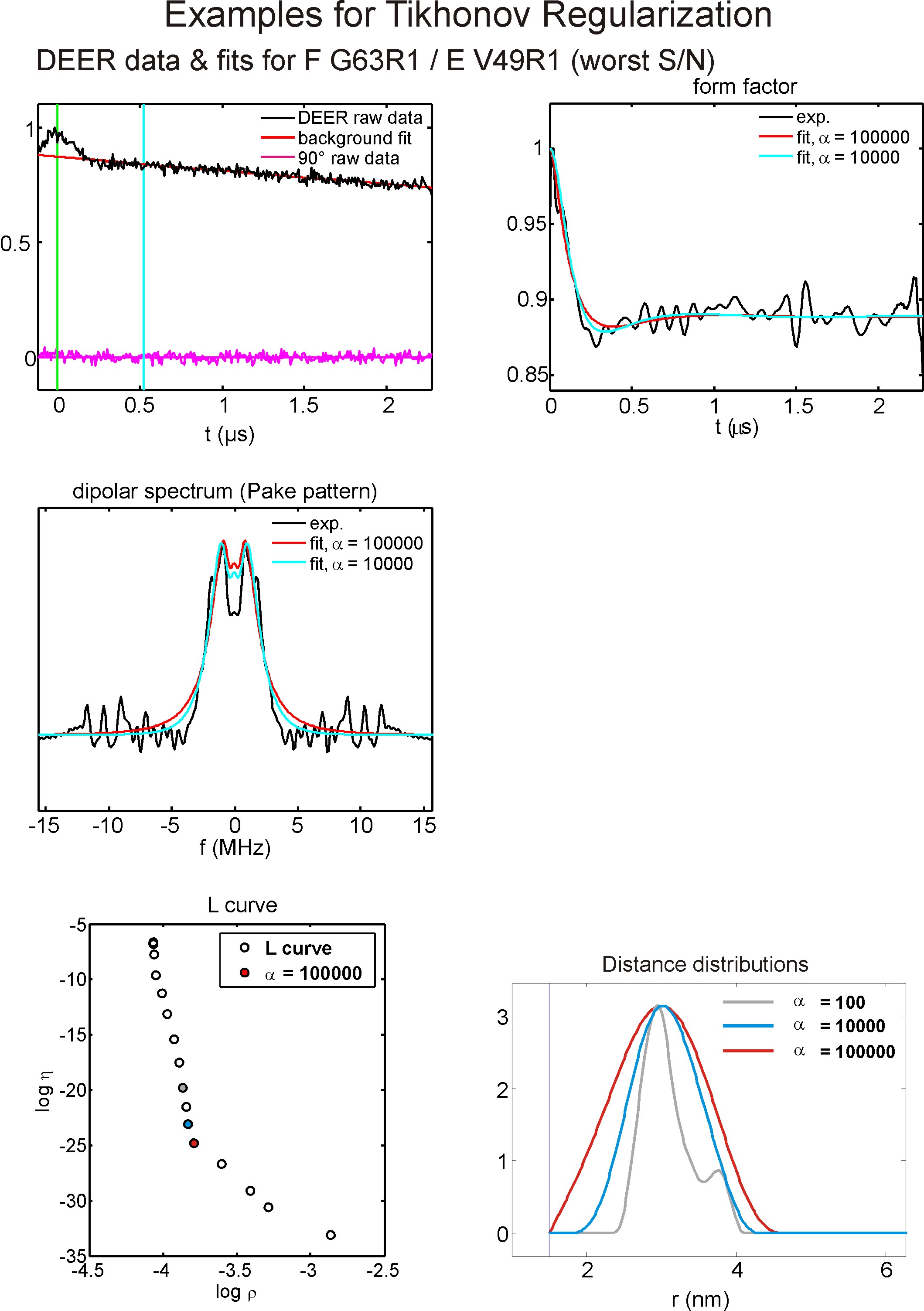


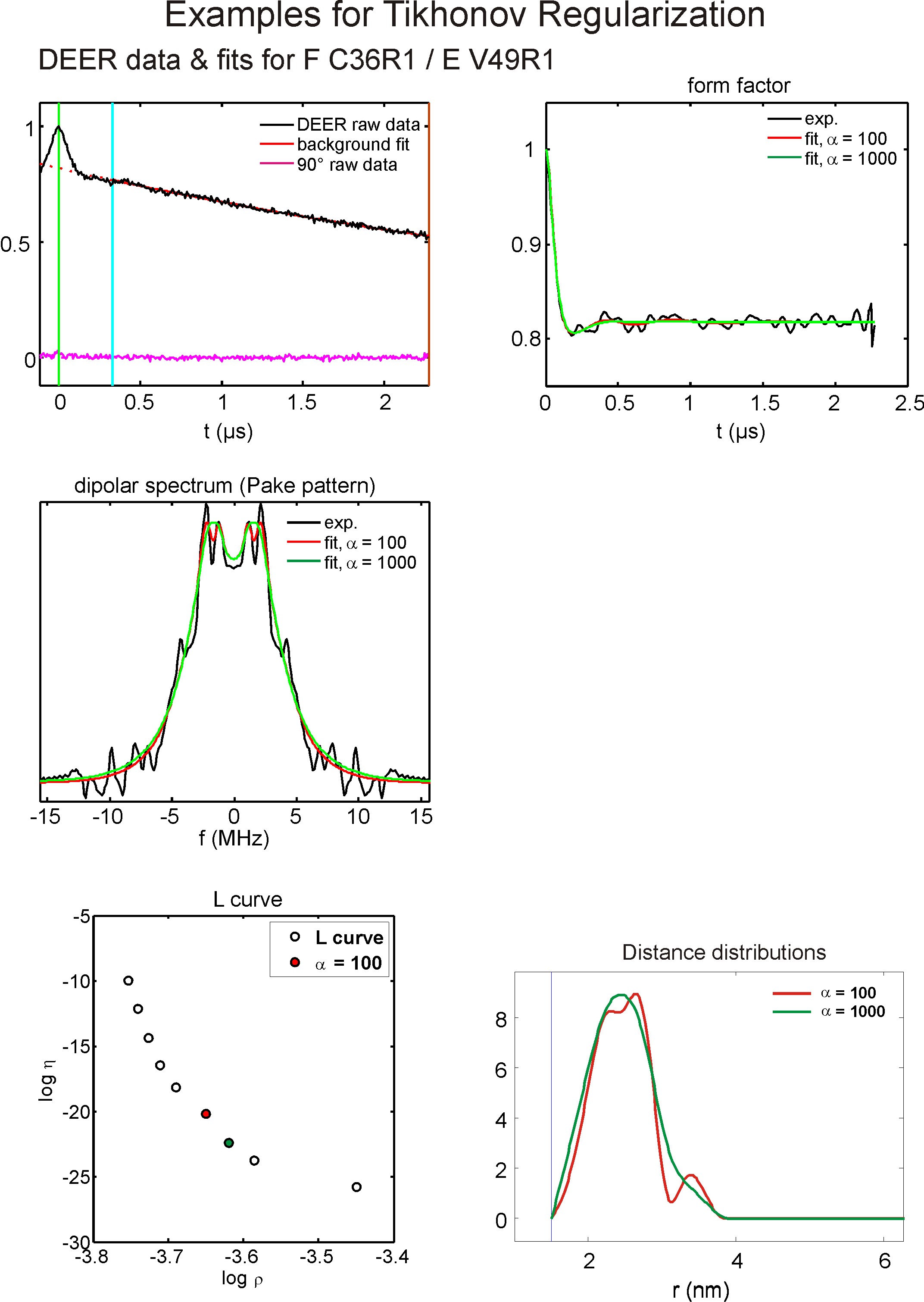


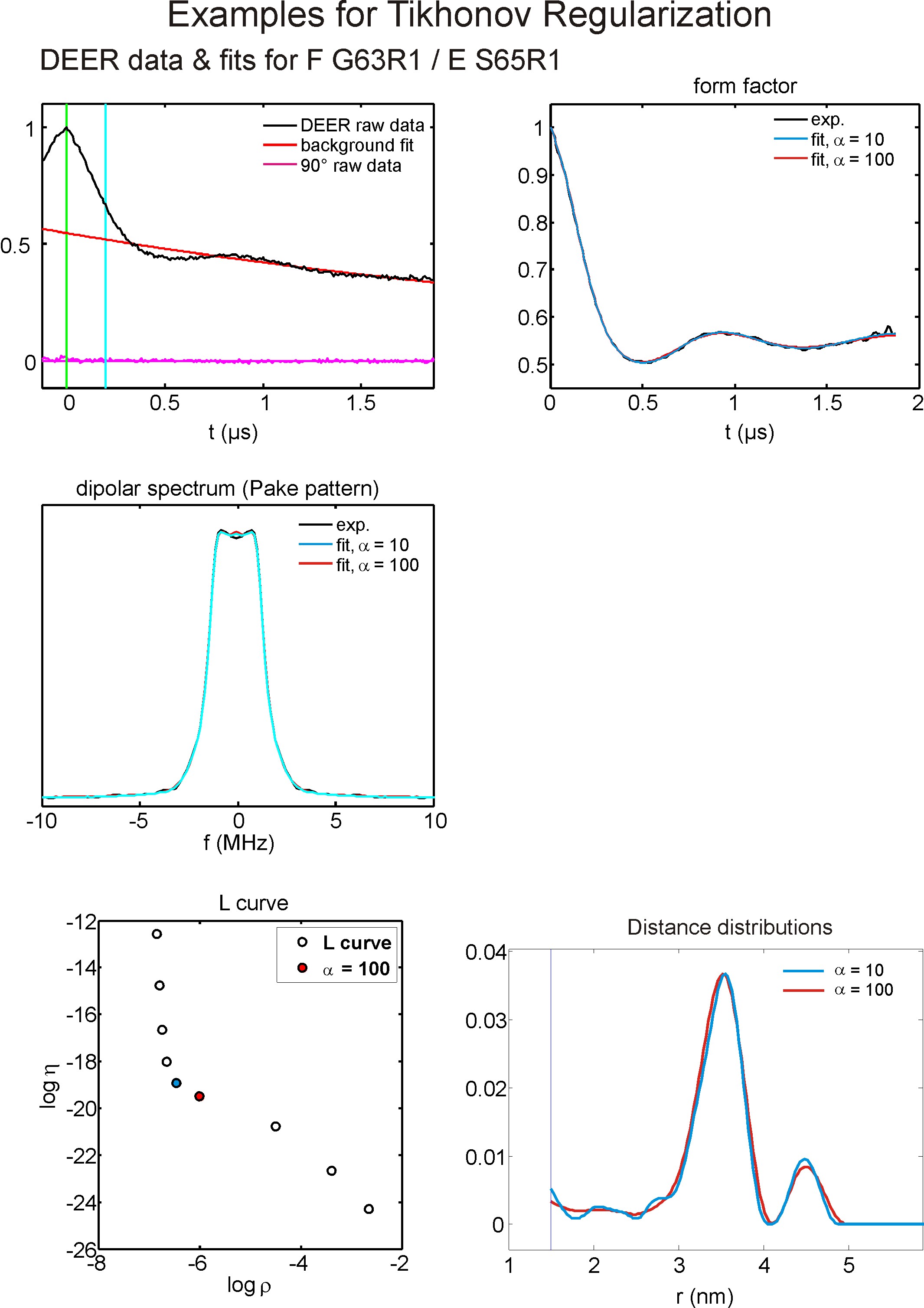


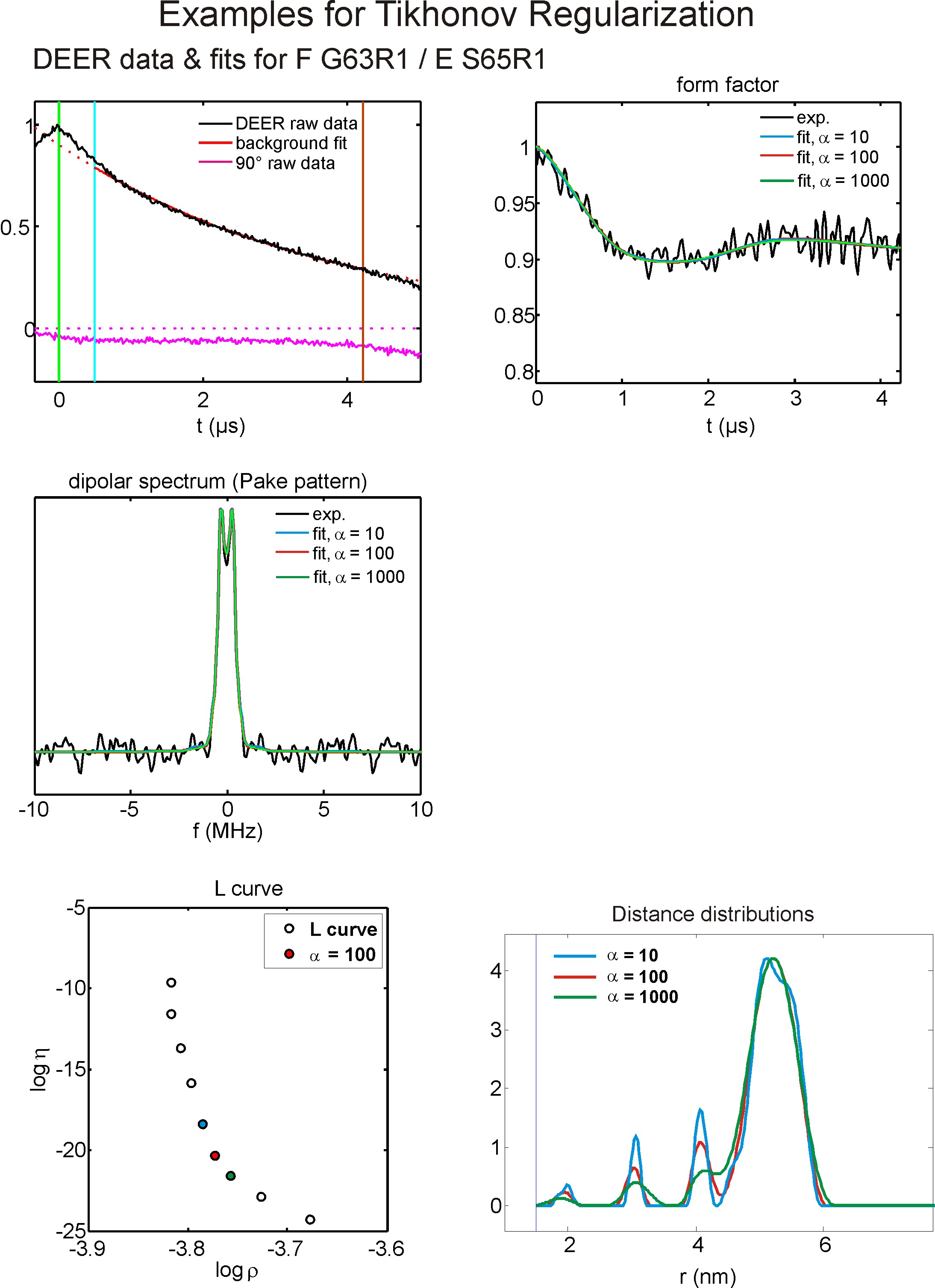


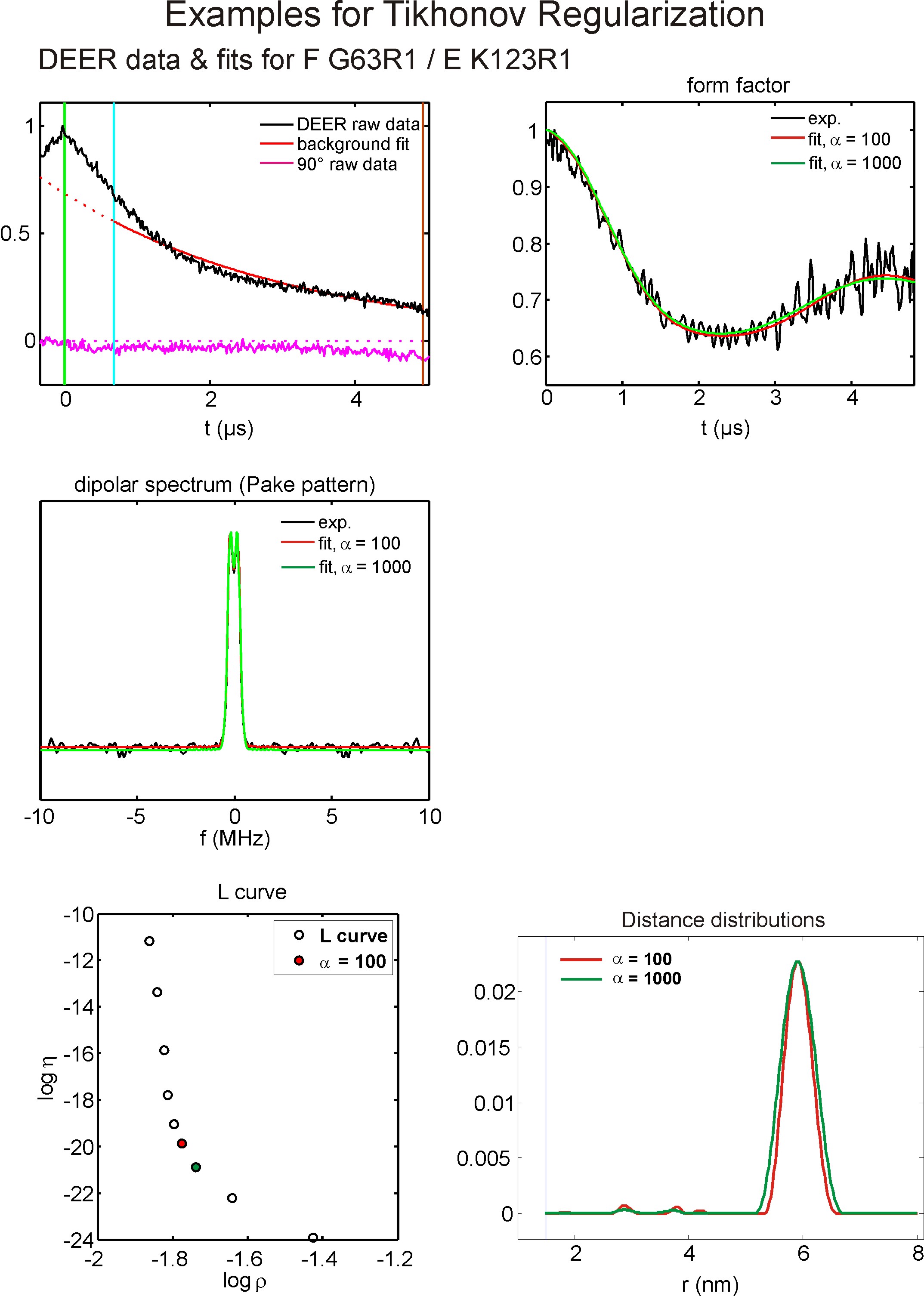

Supplement: Text S1 — Tikhonov regularization and DEER data analyses. A brief description of the theoretical background and procedure of DEER data analysis by Tikhonov regularization, and detailed analyses of the DEER data presented in this paper. (DOC) [file pone.0039492.s009.doc]
