# Supplementary material for: Simulation vs. Reality: A Comparison of In Silico Distance Predictions with DEER and FRET Measurements
Source: PLoS One. 2012 Jun 25;7(6):e39492. doi: 10.1371/journal.pone.0039492 (PMC3382601; doi:10.1371/journal.pone.0039492)
Supplement: Text S2 — Atom type definitions used for MC sampling. Atom types added to the VegaZZ template CHARMM22_PRO for MC sampling of the fluorophores. (DOC) [file pone.0039492.s010.doc]

**Supplementary Text S2: Atom type definitions used for MC sampling**

For the MC sampling with the fluorophores A350 and A488, the atom types listed below were added to the VegaZZ template CHARMM22_PRO using ATDL (Atom Type Description Language) according to the programme manual (chapter “Creating a new template“).

The partial charges and parameters used were the same as in the MD simulations.

**HP H-100 (C-350 (C-350 C-350))**

**CPT C-361 (C-361 C-361 C-360 (C-400))**

**CPT C-361 (C-361 C-361 C-361 (O-260))**

**CPT C-361 (C-361 C-361 O-260 (C-360))**

**CSF C-361 (S-400 C-361 C-361)**

**CA C-360 (O-260 O-100 C-360 (C-360 C-400))**

**CA C-350 (C-350 C-350 (N-350) H-100)**

**CCO C-300 (O-100 C-400 (C-360) N-300)**

**CCO C-300 (O-100 C-361 (C-361 C-361) N-300)**

**CC C-350 (O-100 N-350 (C-400 C-350) C-350)**

**CA C-360 (C-361 C-360 C-400)**

**CA C-360 (C-360 C-360 C-400)**

**CS C-350 (S-200 C-350 C-350)**

**NH2 N-300 (C-361 (C-361 C-361) H-100 H-100)**

**NH2 N-350 (C-350 (O-100) C-350 (O-100) C-400)**

**OES O-260 (C-361 C-360 (O-100 C-360))**

**OES O-260 (C-361 C-361 (C-361 C-361))**

**OC O-100 (C-360 (O-260 C-360))**

**OSF O-100 (S-400)**

**SM S-200 (C-400 C-350 (C-350 C-350))**

**SSF S-400 (O-100 O-100 O-100 C-361)**
